# Reciprocal modulation of mesenchymal stem cells and tumor cells promotes lung cancer metastasis

**DOI:** 10.1016/j.ebiom.2018.02.017

**Published:** 2018-02-23

**Authors:** Giulia Fregni, Mathieu Quinodoz, Emely Möller, Joanna Vuille, Sabine Galland, Carlo Fusco, Patricia Martin, Igor Letovanec, Paolo Provero, Carlo Rivolta, Nicolo Riggi, Ivan Stamenkovic

**Affiliations:** a Experimental Pathology Service, CHUV and University of Lausanne, 1011, Switzerland; b Department of Computational Biology, Unit of Medical Genetics, University of Lausanne, 1011, Switzerland; c Center for Translational Genomics and Bioinformatics, San Raffaele Scientific Institute, IRCCS, Milan20132, Italy; d Dept. of Molecular Biotechnology and Health Sciences, University of Turin, 10126 Turin, Italy; e Department of Genetics and Genome Biology, University of Leicester, Leicester LE1 9HN, UK

**Keywords:** Tumor-associated MSCs, lung cancer, metastasis, GREM1, LOXL2, ADAMTS12, ITGA11

## Abstract

Metastasis is a multi-step process in which direct crosstalk between cancer cells and their microenvironment plays a key role. Here, we assessed the effect of paired tumor-associated and normal lung tissue mesenchymal stem cells (MSCs) on the growth and dissemination of primary human lung carcinoma cells isolated from the same patients. We show that the tumor microenvironment modulates MSC gene expression and identify a four-gene MSC signature that is functionally implicated in promoting metastasis. We also demonstrate that tumor-associated MSCs induce the expression of genes associated with an aggressive phenotype in primary lung cancer cells and selectively promote their dissemination rather than local growth. Our observations provide insight into mechanisms by which the stroma promotes lung cancer metastasis.

## Highlights

• Distinct gene expression profiles distinguish normal lung and tumor-associated MSCs.
• MSCs induce EMT- and hypoxia-related genes in primary tumor cells and promote their metastatic potential.
• A 4-gene T-MSC signature is involved in MSC-induced metastasis promotion.

The tumor microenvironment, which includes mesenchymal stem cells (MSCs) among many other stromal cell types, plays a fundamental role in cancer metastasis. Although MSCs are suggested to participate in tumor progression, most studies thus far have been performed on bone marrow-derived MSCs and cancer cell lines. Using primary human pulmonary MSCs and paired lung cancer cells, we show that tumor cells modulate MSCs to acquire properties, including a four-gene signature, which allow them to promote tumor dissemination. Our results provide insight into the mutual cancer cell-stromal cell modulation that drives tumor dissemination.

## 1 Introduction

Metastases are responsible for 90% of cancer-related deaths, as they are resistant to virtually all currently available forms of therapy, surgical removal being limited to isolated lesions. The complex series of steps, known as the metastatic cascade, which tumor cells capable of generating secondary colonies must complete (Chambers et al., 2002; Gupta and Massagué, 2006; Lambert et al., 2017), led to the notion that metastatic cells possess unique, genetically determined features distinct from those of non-metastasizing cells. However, genetic studies, including large-scale genomic sequencing efforts have failed to uncover metastasis-specific mutations (Garraway and Lander, 2013; Vogelstein et al., 2013). Cancer cells capable of fulfilling all of the requirements for both primary and metastatic growth must therefore reside within the initial tumor mass (Ramaswamy et al., 2003), their metastatic properties being most likely sculpted by epigenetic mechanisms activated by the initiating oncogenic events themselves or by signals delivered by the microenvironment (Wan et al., 2013).

The tumor microenvironment (TME) plays a critical role in metastasis. Tumor growth invariably induces an inflammatory and regenerative response, which recapitulates the features of a wound (Chang et al., 2004), while suppressing the mechanisms that render a wound self-limiting, leading to the analogy of a “wound that never heals” (Dvorak, 1986). Elucidation of the mechanisms by which the microenvironment promotes tumor cell dissemination therefore requires thorough understanding of the wound healing process, including the discrete changes in the stromal cell populations and extracellular matrix (ECM) composition that occur as the process evolves (Schäfer and Werner, 2008).

Host tissue stromal cells provide favorable conditions for tumor cell dissemination by producing cytokines that promote tumor cell survival and growth and by secreting proteolytic enzymes that break down physical barriers to cell migration and release growth factors sequestered within the ECM (Quail and Joyce, 2013). Among stromal cells that promote tumor progression, cancer-associated fibroblasts (CAFs) are held to play the principal role (Kalluri, 2016). However, CAF is a loosely used term and in addition to activated fibroblasts, which typically express α-smooth muscle actin (α-SMA) and are referred to as myofibroblasts, CAFs include a variety of mesenchymal cells at various stages of differentiation and with various degrees of plasticity (Kalluri, 2016; Öhlund et al., 2014). The potent regenerative signals of the TME not only promote cancer cell pluripotency (Taddei et al., 2013) but also recruit stromal cells associated with tissue regeneration (Mcallister and Weinberg, 2014), prominent among which are mesenchymal stem cells (MSCs) (Cuiffo and Karnoub, 2012).

Mesenchymal stem cells are heterogeneous stromal cells defined based on functional and phenotypic criteria, including adherence to plastic under standard culture conditions, expression of selected cell surface markers and lack of lineage-specific markers and the capacity to differentiate into most mesenchymal lineages (Dominici et al., 2006; Kobolak et al., 2016). They were initially identified in the bone marrow (BM) but subsequently found to be recruited to or to reside in most tissues where they contribute to tissue renovation especially in situations of acute and chronic injury. Mesenchymal stem cells display tropism for inflammatory and tumor sites, where their release of a broad repertoire of soluble factors can modulate the immune response and affect tumor cell behavior (Cuiffo and Karnoub, 2012). They can also differentiate *in situ* into a variety of mesenchymal lineages (Bergfeld and DeClerck, 2010; Cuiffo and Karnoub, 2012) and have been suggested to promote tumor metastasis (Karnoub et al., 2007; Koh and Kang, 2012) based on models using BM-MSCs and cancer cell lines (Bergfeld and DeClerck, 2010). Only recently has interest begun to shift toward the influence of human tumor associated-MSCs on cancer progression (Shi et al., 2017; Sun et al., 2014).

We addressed the role of MSCs on the growth and dissemination of lung cancer, the leading malignancy in terms of lethality worldwide. More than 85% of lung cancers are non-small cell lung carcinomas (NSCLC), which are subdivided into adenocarcinoma (AC), squamous cell carcinoma (SCC) and large cell carcinoma, that comprise about 50%, 40% and <10% of NSCLC, respectively. NSCLC respond poorly to conventional chemotherapy and although targeted therapy has been successful in prolonging survival in a minority of cases (Alamgeer et al., 2013; Hirsch et al., 2017), the current 5-year survival of NSCLC patients is lower than 20% (Chen et al., 2014). Small cell lung carcinomas (SCLC), which comprise the remaining 15% of lung cancers are even more aggressive than NSCLC with extremely high metastatic proclivity and 5-year patient survival below 7% (Semenova et al., 2015).

Using patient-derived lung cancer samples removed at surgery, we compared the effect of tumor-associated MSCs (T-MSCs) to that of normal adjacent lung tissue-derived MSCs (N-MSCs) on the behavior of autologous primary lung cancer cells. Injection of the tumor cells with paired T- or N- MSCs into the subcapsular renal compartment of NOD-SCID- common-γ-KO (NSG) mice revealed that T-MSCs promoted multi-organ metastasis without augmenting local growth of tumor cells, which alone displayed low metastatic proclivity. Although T- and N-MSCs displayed different gene expression profiles, *in vitro* experiments revealed that tumor cells and TME factors participate in promoting N-MSC transition toward a T-MSC phenotype. Conversely, MSCs caused tumor cells to upregulate genes associated with tumor dissemination. Reconstitution of N-MSCs with four genes, *GREM1*, *LOXL2*, *ADAMTS12* and *ITGA11* that contributed to the T-MSC phenotype increased their ability to promote primary tumor cell dissemination. Our observations provide insight into mechanisms by which MSCs selectively promote cancer metastasis independent of their immunosuppressive functions.

## 2 Experimental Procedures

### 2.1 Isolation and Characterization of MSCs and Tumor Cells

#### 2.1.1 MSCs

Primary fresh tumor tissues and macroscopically normal adjacent tissues were obtained from 5 SCC, 3 AC and 2 SCLC patients (Table 1) by surgical resection at Centre Universitaire Hospitalier Vaudois (CHUV) with patient signed informed consent according to the guidelines of the Ethic committee of Canton de Vaud (project authorization n° 131/12) and conforming to standards indicated by the Declaration of Helsinki. MSC proportions in tumor and normal bulk tissues were assessed by flow cytometry among CD45^−^CD34^−^CD20^−^CD14 (Lin^−^) cells using the MSC phenotyping kit (Miltenyi Biotec Cat# 130-095-198) (see Supplemental Experimental Procedures). N- and T-MSCs were obtained after mechanical and enzymatic tissue disruption in IMDM (Gibco) supplemented with Collagenase II and IV (0,5 mg/ml, Gibco) and DNAse (0,1 mg/ml, Roche) for 2 h at 37 °C and passed through a 100 μm cell strainer. The resulting single cell bulk was cultured one night in MSC medium: IMDM + GlutaMAX (Gibco) supplemented with 10% fetal bovine serum (FBS) (PAN Biotech), 1% penicillin streptomycin (PS, Gibco), 1% non-essential amino acids (NEAA, Gibco) and 10 ng/ml platelet derived growth factor (PDGF, Prospec). The following day, the whole medium was changed and only adherent cells were kept. When reaching 80% confluence, cells were split 1:4–1:6 using trypsin-EDTA 0.25 mg/ml (Lonza, USA) and kept in culture in MSC medium. MSCs phenotype was analyzed by flow cytometry using anti-human CD90-FITC (Fluorescein isothiocyanate; Milteny Biotec Cat# 130-095-198), CD166-PerCP-Cy5.5 (Peridinin Chlorophyll Protein Complex Cyanine; BD Pharmingen Cat#562131), CD105-PE (Phycoerythrin; Milteny Cat# 130-095-198), CD73-APC (Allophycocyanin; Milteny Biotec Cat# 130-095-198), CD44-APC-H7 (BD Pharmingen Cat#560532), CD45-AlexaFluor700 (BD Pharmingen Cat#560566) antibodies and vimentin (Dako #M0725) and alpha-SMA (Abcam #ab5694) expression by immunohistochemistry (IHC) (for detailed information see Suppl. Exp. Procedures). The differentiation potential in adipocytes, osteocytes and chondrocytes was assessed (see Suppl. Exp. Procedures). BM-MSCs were isolated from the iliac crest of 3 healthy donors (Fig. S1C; project authorization n° 131/12) and used as control. For all experiments, cells were used between passage 2 and 9.

**Table 1:** Patient characteristics.

Patient	Tumor type	Tumor stage^f^	Neoadjuvant	Gender	Age
#12	Moderately differentiated SCC^a^	pT2b N0 Mx	No	F	79
#16	SCLC^b^	pT3 N1	NA^g^	F	58
#19	AC^c^	pT2a N0	No	M	62
#20	AC	pT4 N2	No	M	64
#21	Poorly differentiated SCC	pT2a pN0	No	F	64
#26	Composite carcinoma (SCLC 60%, NELC^d^ 30%, NSCLC NOS^e^ 10%)	pT2b N1 Mx	No	M	68
#27	Moderately differentiated SCC	pT3 pN0 Mx	No	M	70
#28	AC	pT1b pN0	No	F	75
#29	Moderately differentiated SCC	pT2a pN0	No	M	74
#32	Poorly differentiated SCC	pT2a pN0	No	M	84

a SCC: Squamous cell carcinoma.

b SCLC: Small cell lung carcinoma.

c AC: Adenocarcinoma.

d NELC: neuroendocrine lung carcinoma.

e NSCLC NOS: non-small cell lung cancer not otherwise specified.

f TNM classification: p: pathological classification; T: primary tumor; N: lymph node; M: metastasis; x: not assessed histologically.

g NA: not available.

#### 2.1.2 Tumor Cells

Primary tumor cells from patients 21, 26, 32 were obtained culturing single cell tumor bulk as spheres in ultra-low attachment flasks (Corning, Falcon) in KO medium: IMDM + GlutaMAX completed with 20% knockout serum (Gibco), 20 ng/ml leukemia inhibitory factor (LIF) (Prospec), 20 ng/ml recombinant human (rh) epidermal growth factor (EGF) (Prospec), 20 ng/ml fibroblast growth factor (FGF) (Prospec) and PS 1%. The clonogenic potential was assessed as well as the expression of tumor markers expressed by parental tumors (see Suppl. Exp. Procedures).

### 2.2 Xenotransplants and Metastasis Quantification

#### 2.2.1 Cell Injection

Experimental protocols involving mice were approved by the Veterinary Service of the Canton of Vaud (Etat de Vaud, Service Vétérinaire), under authorization number VD2488.1. For all experiments sphere-forming tumor cells were injected as single cell beneath the renal capsule (left kidney) of NOD-SCID- common-γ-KO (NSG) mice. All mice were females 4–7 week old at the time of injection. Each injection was performed using a Hamilton syringe in 20 μl of volume and cell numbers are reported in Fig. S1E. Mice were sacrificed when tumors reached 1 cm^3^ or when the animals showed signs of distress. Otherwise they were observed for six months after injection. Tumors were weighed and fixed in PFA 4%. Organs (spleen, right kidney, lung and liver) were fixed and paraffin embedded (Leica ASP200S) for subsequent metastasis quantification.

#### 2.2.2 Metastasis Quantification

Metastases were quantified on 3 μm thick coronal organ sections stained with hematoxylin and eosin (HE). Images of organ slices were then acquired by the NanoZoomer Digital Pathology slide scanner (NDP, Hamamatsu) and metastases analyzed by imaging in a blind manner using the NDP.view 2 software. Areas of manually drawn circles around organs and metastasis (as shown in Fig. S2A) were automatically calculated by the software and data exported in .csv Excel files. For each organ, the proportion (%) of the total metastatic area was calculated as: (total metastatic area) ∗ 100 / (total organ area). For each mouse, the percentage of the total metastatic area was assessed as: (sum of the total organ (spleen + lung + kidney + liver) metastatic areas) ∗ 100 / (sum of the total organ areas). Numbers of individual metastases were also counted and expressed as the number of metastases per organ or as a sum of the four organs. The proportion of mice bearing 0, 1–2 or 3–4 metastatic organs was calculated within each group.

#### 2.2.3 Assessment of Primary Cell Tumorigenic Potential

Tumor cells from patients 21, 26 and 32 were first injected alone (n = 3 mice/patient sample) in growth factor-reduced (GFR) matrigel (Becton Dickinson AG) to assess their tumorigenicity. To exclude the presence of tumor contaminating cells, T-MSCs from patients 21, 26 and 32 were injected alone (n = 3 mice/patient sample) at high numbers (Fig. S1E) in GFR-matrigel.

#### 2.2.4 Tumor Cell Co-injection With Primary MSCs

For co-injection experiments, primary tumor cells were injected with MSCs in IMDM at a 1:1 ratio in the numbers reported in Fig. S1E. For 21 and 32 tumor cell co-injection with MSCs, 2 independent experiments were performed that gave similar results and allowed us to pool the data together. In total, for tumor 21, 11 mice were injected in the “control” group (ctrl, tumor cells alone), 5 mice in “+BM-MSC” group, 14 mice in “+T-MSC” groups and 15 mice in “+N-MSC” (corresponding to tumor cells co-injected with BM-, T- or N-MSCs respectively). For tumor 26, 3 mice per group were injected; however, 1 mouse in “+BM-MSC” group died for unrelated reasons and was excluded from the study. For tumor 32, 14 mice were injected in total in the “ctrl” group, 5 mice in the “+BM-MSC” group, 14 mice in the “+T-MSC” group and 15 mice in the “+N-MSC” group. Mice without tumors (n = 3: 1x21 + BM-MSC, 1x32ctrl, 1x32 + N-MSC) were excluded from all analyses and graphs but included in the tumorigenic potential count reported in Fig. 2.

#### 2.2.5 Tumor Cell Co-injection With Engineered-MSCs

In the 26 tumor cell/engineered-MSCs co-injection experiment, 2000 tumor cells were injected at a 1:1 ratio with the total number of MSCs: N- or T-MSCs expressing only the *Emerald* reporter gene (N-MSC^*EM*^ and T-MSC^*EM*^, respectively), or a bulk of N-MSCs overexpressing either *GREM1*, *LOXL2*, *ITGA11* or *ADAMTS12* (500 cells of each; N-MSC^*GAIL*^). 6 mice were injected with 26 tumor cells +N-MSC^*EM*^ cells, 7 mice with 26 tumor cells +N-MSC^*GAIL*^ cells and 7 mice with 26 tumor cells +T-MSC^*EM*^ cells. In this experiment, tumor growth was also monitored by ultrasound imaging. See “N-MSC overexpression of selected genes” section for more details about engineered cell preparation.

#### 2.2.6 Ultrasound Imaging

Tumor volume was calculated by *V* = 4/3 p(*D*d × *D*s × *D*t)/8, where *D*d corresponds to tumor height, and *D*s and *D*t to tumor lengths measured in long- and short-axis views, respectively.

### 2.3 MSC Expression Profile Analysis by Microarray

Total RNA was extracted from MSC samples between passage 5 and 6 using TRIzol reagent (Ambion, Life Technologies, USA) and following standard manufacturer protocol. Affymetrix Human Gene 1.0 ST arrays was used to compare the gene expression profile between N- and T-MSCs. Detailed information on technical procedure are reported in Suppl. Exp. Procedures. Background correction, normalization, and probe summarization were done using the Robust Multichip Average (RMA) Method implemented in the Expression Console software Version (v) 1.4.1 (Affymetrix). Filtering, hierarchical clustering (HCL), principal component analysis (PCA), and statistical analysis of log_2_ transformed expression data were conducted using the Qlucore Omics Explorer v 3.0 (Qlucore AB, Lund, Sweden). For PCA based on Pearson correlation matrix, the data were normalized through the settings mean = 0 and σ = 1 and variance filtered on the basis of the ratio *σ*/*σ*_max_. HCL was conducted on the basis of Euclidean distance (samples) and Pearson correlation (genes). Variables were collapsed (only unique gene symbols) and calculated as average gene expression value. Before the downstream analyses, the following probe collections were removed from the normalized data; miR BASE, Ensembl ncrna, ChrM and ChrUn. Data were pre-filtered with a fixed variance filter of 0.128 (σ/σ_max_) giving a projection score 0.41, with a good representation of the data. These settings captured 70% of the total variance of the dataset (compared to a random dataset of the same size) by 3926/19260 variables. Differentially expressed genes were identified using correlation-matrix based PCA in combination with paired t-test with eliminating factor the patient id number and q-value (false discovery rate, FDR) cut-off 0.2. The Benjamini–Hochberg method (Benjamini and Hochberg, 1995) was used for error correction (*q*-value calculation).

### 2.4 RNA Extraction, cDNA Synthesis and qRT-PCR

For microarray validation, total RNA was extracted from MSC samples using TRIzol reagent. For N-MSC co-culture with tumor cells and cytokine treatment, total RNA was extracted using the RNeasy mini Kit (Qiagen), following the standard manufacturer protocol. For each sample, cDNA was synthesized by reverse transcription using M-MLV Reverse Transcriptase (Promega) according to manufacturer instructions. Levels of gene expression were determined using the 2-ΔΔCT methods (Livak and Schmittgen, 2001) and samples analyzed in triplicates. Quantitative RT-PCR amplification was performed using TaqMan Universal PCR mastermix or SYBR® Green mix (Applied Biosystems). SYBR® Green primer sequences for the quantification of *ADAMTS12*, *BST2*, *CHI3L1*, *FIGF*, *GJA1*, *GREM1*, *IFITIM*, *IL6*, *ITGA11*, *LOX*, *LOXL2* and *MX2* are listed in the table on Suppl. Exp. Procedures section. *PP1A* (protein phosphatase 1; Applied Biosystems, Hs99999904_m1), *18s* (Applied Biosystems, Hs99999901_s1), *GAPDH* or *TBP* (SYBR® Green) were used as housekeeping genes. For microarray validation, for each patient, data were normalized on N-MSC expression levels (fold change = 1). For BM-MSC comparison with N- and T-MSC gene expression levels, data were normalized on expression levels of N-MSCs from patient 12 (fold change = 1). For N-MSC co-cultures with tumor cells or treatment by cytokines, expression levels were normalized on N-MSCs cultured in control condition (fold change = 1).

### 2.5 Secretome Analysis

Secretome of N- and T-MSCs from patients 12 and 21 was assessed in concentrated supernatants from cells cultured for 24 h in IMDM without phenol red supplemented with PS1% and PDGF 10 ng/ml. Samples were concentrated, digested with trypsin and analyzed on a high resolution hybrid LTQ Orbitrap Velos mass spectrometer (Thermo Fisher Scientific, Bremen, Germany) coupled to a nano-liquid chromatography system. Repeated analysis of 21N- and 21T-MSC secretomes together with BM1 and BM3 samples (Fig. S3B) were prepared and processed similarly, with the only difference that MS analysis was done on a Fusion Tribrid Orbitrap instrument (Thermo Fisher Scientific, Bremen, Germany). More details on sample preparation and analysis can be found in Suppl. Exp. Procedures.

### 2.6 N-MSC Expression Analysis After Tumor Cell Co-culture and Cytokine Treatment

#### 2.6.1 Co-culture

N-MSCs and tumor cells from patients 21, 26 and 32 were co-cultured in transwell conditions at different N-MSC:tumor cell ratios: 2:1, 1:1, 1:2 and 1:5. Co-cultures were analyzed after 3, 5 and 7 days. N-MSCs (20.000 cells/well) were seeded at passage 5 onto six-well plates (Costar, Corning incorporated). Tumor cells were seeded into 1,0 μm-pore insert of PET-membrane (Corning, Falcon) at different amounts according to the four different ratios. Controls were T- and N-MSCs cultured alone.

#### 2.6.2 TGF-β1 and IL-6 Treatment

21, 26 and 32 N-MSCs (20.000 cells/well) were seeded in six-well plates and treated for 3, 5 and 7 days either with IL-6 (10 ng/ml, Sigma), TGF-β1 (1 ng/ml or 10 ng/ml, Miltenyi Biotec) or a combination of the two in MSC medium. Controls were untreated N-MSCs.

#### 2.6.3 Co-culture in Presence of TGF-β1 Treatment

N-MSCs and tumor cells from patients 21, 26 and 32 were co-cultured in transwell conditions as described above at 1:1 and 1:5 N-MSC:tumor cell ratios in the presence of TGF-β1 10 ng/ml. Co-cultures were analyzed after 3, 5 and 7 days. Controls were N-MSCs cultured alone in absence of TGF-β1.

For all experiments, cells were cultured in MSC medium, half of which was refreshed at days 3 and 5. At the end of co-culture or treatment, N-MSCs were harvested, snap frozen and stored at -80 °C until RNA extraction. Gene expression was assessed by qRT-PCR as previously described and data normalized to corresponding expression levels in N-MSCs cultured alone and/or untreated (fold change = 1).

### 2.7 3D Spheroid Invasion Assay

Tumor cells from patients 21, 26 and 32 were stained with CellTrace CFSE 10 μm (Carboxyfluorescein succinimidyl ester; Molecular Probes; Cat# C34554) according to the manufacturer's instructions. CFSE-labeled tumor cells (2500 cells/45 μl drop) were cultured in hanging drop plates (HDP Perfecta3D 96-Well Hanging Drop Plates; 3D Biomatrix) according to the manufacturer's instructions for 60 h in the presence or absence of paired N- or T-MSCs at a 1:1 MSC:tumor cell ratio to allow spheroid formations. N- and T-MSCs alone (2500 cells/45 μl drop) were cultured under the same conditions and used as supplemental controls for MSC migration. All conditions were performed in quadruplicate. After 60 h, spheroids were transferred by centrifugation (5 min, 180 g, at 4 °C) to a 96-well U-bottom (TPP) plate filled with pre-chilled invasion matrix 50 μl/well (Cultrex Invasion Matrix; Amsbio; Cat# 3500-096-03) according to the manufacturer's instructions. The 96-well U-bottom plates containing the spheroids were then incubated for 1 h at 37 °C to promote polymerization of the matrix. After polymerization, 100 μl/well of KO medium were added and plates kept at 37 °C for 4 days. Medium was refreshed at 48 h by adding 50 μl/well of KO medium. For each spheroid, images were taken by light and fluorescent microscopy at day 0 and every 24 h thereafter using a 4× objective. Lighting and focus were adjusted to provide maximal contrast between the 3D structure and background.

### 2.8 Tumor Cell Co-cultures With MSCs and RNA Sequencing

#### 2.8.1 Co-culture

21 and 32 tumors were dissociated to single cells and seeded onto ultra-low attachment six well plates (Costar, Corning) at 50.000 cells/well and maintained in transwell culture for 5 days with paired N- or T-MSCs at a 1:1 tumor:MSC cell ratio. For both tumor cell types, a second independent experiment was performed using 29 MSCs. MSCs were seeded into 1,0 μm-pore inserts of PET-membrane (Corning, Falcon). Controls were tumor cells cultured alone. Cells were cultured in KO medium, half of which was refreshed at day 3. After removal of the upper chamber containing MSC cells, tumor cells were harvested, snap frozen and stored at -80°C until RNA extraction with miRCURY RNA isolation kit (Exiqon) following manufacturer instructions including a DNAse step with RNAse-Free DNase set (QIAGEN). RNA was stored at -80°C until RNA-seq analysis.

#### 2.8.2 RNA-seq Analysis

The TruSeq mRNA stranded kit from Illumina was used for the library preparation. Pools of 6 libraries were loaded at 8.5 pM for clustering on a Single-read Illumina Flow cell. Reads of 100 bases were generated using the TruSeq SBS HS v3 chemistry on an Illumina HiSeq 2500 sequencer. PCA analysis was done with R v3.3.2 using *limma* package to remove batch and sample effects and *stats* package for the analysis. The differential expression analysis was performed with edgeR v3.10.5. The differentially expressed genes were defined as those with P-value < 0.05, fold change (FC) > 2 and detectable expression. Gene ontology analysis was performed using GSEA software and Molecular Signature Database (MSigDB) (Subramanian et al., 2005), http://software.broadinstitute.org/gsea/index.jsp. For more detailed information, see Suppl. Exp. Procedures.

### 2.9 Transwell Invasion Assay

Transwell (TW) invasion of CFSE-labeled tumor cells from patients 21 and 32 was assessed through a matrigel-coated membrane in response to different culture conditions: the presence of N- or T-MSCs (in IMDM-FBS 10%) in the lower invasion chamber or of IMDM medium supplemented or not with 10% FBS. After overnight culture, images of invading tumor cells were taken in the lower chamber and on the membrane by light and fluorescent microscopy using a 4× objective. For more detailed information, see Suppl. Exp. Procedures.

### 2.10 Microscopy

#### 2.10.1 MSC Differentiation and IHC Assays

Images were taken with a Nikon Eclipse E800 digital camera DXM1200 with a resolution of 1280 × 1024 at 20× or 40× magnification and analyzed with the ACT-1 (v.2) software

#### 2.10.2 Spheroid and TW Invasion Assays

Images were taken with a Olympus IX53, digital camera U-HGLGPS, with a resolution of at 4× magnification, and analyzed with the cellSens Entry (v1.13) Olympus software.

### 2.11 N-MSC Overexpression of Selected Genes

First, we cloned the *Emerald* (*EM*) gene (Invitrogen) into a lentiviral plasmid (pLIV) derived from the pLVTH backbone (addgene) to express the puromycin resistance gene (Puro-r). *GREM1*, *LOXL2*, *ADAMTS12* and *ITGA11* genes were then cloned in the resulting pLIV_Puro_2A_Em backbone. Detailed information about cloning, virus production and titration are reported in Suppl. Exp. Procedures.

N- and T-MSCs from patient 26 were infected at passage 5 by two rounds of infection using Polybreen 8 ng/ml (Sigma) with an overnight pause in MSC medium and at a 0.5 MOI (multiplicity of infection). Antibiotic selection (Puromycin, 2 μg/ml) was added 48 h after the second infection and kept for 4 days. N-MSCs were infected either with EM control virus (N-MSC^*EM*^) or with single-gene overexpressing viruses. For mice injection, equal numbers (1/4 each) of single-gene overexpressing N-MSCs were taken and injected as a bulk (N-MSC^*GAIL*^). T-MSCs were infected only with EM control virus (T-MSC^*EM*^). Gene overexpression was assessed by qRT-PCR and cells taken for mice injection at passage 3 after infection.

### 2.12 Statistical Analysis

Statistical tests and graphics were generated by Prism version 7.03 (GraphPad Software Inc.). Nonparametric paired Wilcoxon test was used to compare percentages of MSCs in normal and tumor tissue (*, P ≤ 0.05). For mouse injections, tumor weights and metastasis quantification were analyzed using the nonparametric Kruskal–Wallis (K–W) test with Dunn's multiple comparison test to compare each group (tumor cells co-injected with MSCs) with the control group (tumor cells alone). For 26 tumor cell co-injection with engineered MSCs, 26 tumor cells injected with N-MSC^*EM*^ were considered as control group. When significant, adjusted p-values from Dunn's test were reported in the figure and indicated in the graphs as * or ** according with their degree of significance (**, P ≤ 0.01). The correlation between total number of metastasis and metastatic area was assessed using non parametric Spearman correlation test: r and P-values are reported on the graphs. For qRT-PCR data for microarray validation, multiple t tests were used to compare median expression levels between N- and T-MSCs for each patient. Gene expression levels between BM-, N- and T-MSCs were compared using the nonparametric Kruskal-Wallis (K-W) test with Dunn's multiple comparison test. For N-MSC co-cultures with tumor cells and treatment by cytokines, multiple t tests were used to compare each group to N-MSC control group. Significant differences were indicated as *, **, *** or **** according to level of significance (***, P ≤ 0.001; ****, P ≤ 0.0001). Not significant P-values (P < 0.1) were reported and indicated as “ns” (not significant).

### 2.13 Accession Numbers

The array data are deposited in the NCBI Gene Expression Omnibus (GEO) database and accessible through the series accession number GSE104636. RNA-Seq data have been deposited in the NCBI Gene Expression Omnibus (GEO) database and accessible through the series accession number GSE104858.

## 3 Results

### 3.1 Characterization of MSCs and Primary Tumor Cells From Patients With Lung Carcinoma

**Fig. 1:**
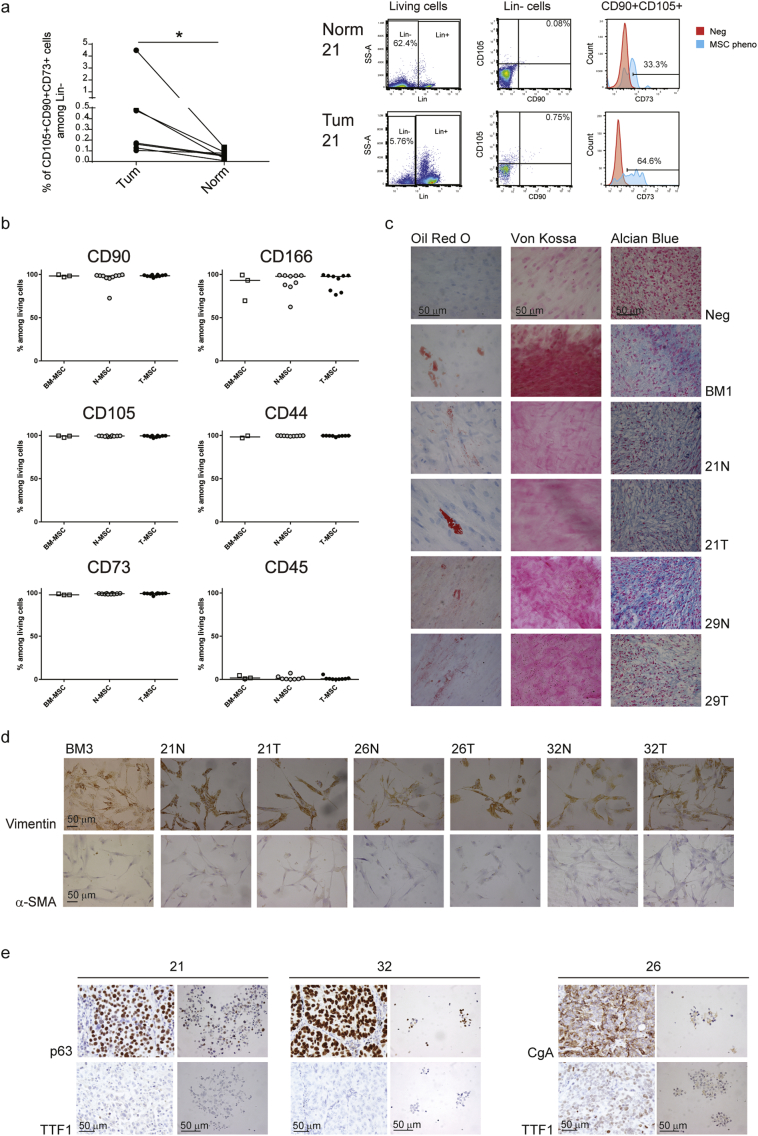
Primary MSC and tumor cell characterization. (A) On the left, proportions of MSC-like (CD105^+^CD90^+^CD73^+^) cells among Lin^−^ cells (CD45^−^CD34^−^CD20^−^CD14^−^) in paired normal and tumor lung tissues (n = 7) assessed by flow cytometry after tissue dissociation. Statistical significance was determined by Wilcoxon matched-pairs signed rank test; P-value is indicated as * according to the level of significance (P < 0.05). On the right, dot plots and histograms from one representative patient (21) depicting the sequential gating strategy on living, Lin^−^ and CD90^+^CD105^+^ cells for the assessment of MSC-like cell proportions in normal and tumor tissues. Percentages of Lin^−^, CD90 and CD105 double positive cells, and CD73^+^ cells among the parental population are reported. CD73 expression by CD90^+^CD105^+^ cells (blue) was calculated using unstained living cells as negative control (red). (B–D) *In vitro* characterization of MSC cell cultures: N- and T-MSCs from lung carcinoma patients (n = 10) were compared with BM-MSCs from healthy donors (n = 3). (B) The expression of MSC surface markers was calculated by flow cytometry and reported as percentages among living cells. Horizontal lines represent medians. (C) Differentiation potential into adipocytes (Oil Red O), osteocytes (Von Kossa) and chondrocytes (Alcian Blue) of MSCs from one representative donor (BM1) and patients 21 and 29. (D) Vimentin and alpha-SMA expressions were assessed immunohistochemically on BM3 and N- and T-MSCs from patient 21, 26 and 32. (E) Sphere-forming tumor cells (right pictures) from 21, 32 and 26 patients were characterized for the expression of tumor markers and compared with tumors of origin (left pictures). (C–E) Scale bar = 50 μm. See also Fig. S1.

Freshly removed untreated primary lung cancer samples and normal adjacent tissues were collected from ten patients diagnosed with SCLC (n = 2), AC (n = 3) or SCC (n = 5) at different stages of progression (pT1bN0-pT4N2) but without distant metastasis (Table 1). Mesenchymal stem cell content was assessed by flow cytometry after tissue dissociation into single cells. Although their percentages were small (ranging from 0.007 to 4.49% among lineage-negative (Lin^−^) cells), MSCs were significantly more abundant in tumor than in normal tissues (P = 0.0156; Figs. 1A and S1A), irrespective of the tumor subtype or the abundance of inflammatory and immune cell infiltrates (considered as Lin^+^ cells, Fig. S1B). Following separation from normal and tumor tissues, the T- and N- stromal cell phenotype was compared to that of BM-MSCs from three healthy donors (BM1 to 3 aged 51, 54 and 78 respectively at the time of surgery; Fig. S1C). Cell surface expression of the MSC-associated markers, CD90, CD166, CD105, CD44 and CD73 (Dominici et al., 2006; Kobolak et al., 2016) was comparable among the three populations of cells (Fig. 1B). Similar to BM-MSCs, N- and T-MSCs (between passage 3 and 7 in culture) could differentiate into adipocytes, osteocytes and chondrocytes (Fig. 1C), with some variability most likely due to patient and donor age (Mijung Kim et al., 2012a; Stolzing et al., 2008). All MSC samples expressed vimentin but were negative for α-SMA (Figs. 1D and S1D), indicating their distinction from myofibroblasts (Kalluri, 2016; Öhlund et al., 2014). Tumor cell presence among the MSCs was excluded by verifying that their karyotype was normal (data not shown) and that even high numbers (ranging from 100000 to 300000 cells per mouse) of T-MSCs injected beneath the renal capsule of NSG mice failed to generate tumors after a six month follow-up (Fig. S1E).

Bulk tumor cell culture in low-adherence conditions in the absence of serum, allowed isolation and expansion of primary tumor cells from two patients (21 and 32) diagnosed with poorly differentiated SCC and one patient (26) with a composite tumor with a 60% SCLC component (Table 1). Tumor cells and paired MSCs will be referred to according to the numbers attributed to the patient samples (21, 26 and 32) they were derived from. Tumor cells were cultured as spheres and assessed for conservation of parental tumor markers. Consistent with their SCC identity, 21 and 32 spheres were positive for p63 and negative for TTF-1 (Gurda et al., 2015; Kalhor et al., 2006; Noh and Shim, 2012) (Fig. 1E). Spheres from tumor 26 were positive for chromogranin A (CgA) and TTF-1 expression (Van Meerbeeck et al., 2011), reflecting the SCLC/neuroendocrine phenotype of their tumor of origin (Fig. 1E). To assess their self-renewal, spheres were dissociated, plated as single cells and monitored for colony formation (Fig. S1F). Cells from the three tumors displayed comparable clonogenic potential with mean formation of 29.9, 30.3 and 25.3 spheres/100 wells by cells from patients 21, 26 and 32, respectively. Injection of low cell numbers (ranging from 1000 to 5000 cells per mouse; Fig. S1E) from all three cultures beneath the renal capsule of NSG mice resulted in tumor formation but with different kinetics, requiring subsequent adjustment of the number of cells injected to synchronize tumor engraftment (Fig. S1E). Successful isolation, of primary lung MSCs along with the corresponding lung cancer cells from three patients, provided us with unprecedented means to probe the functional relationship between MSCs and autologous cancer cells.

### 3.2 T-MSCs Enhance Metastasis of Paired Primary Cancer Cells

**Fig. 2:**
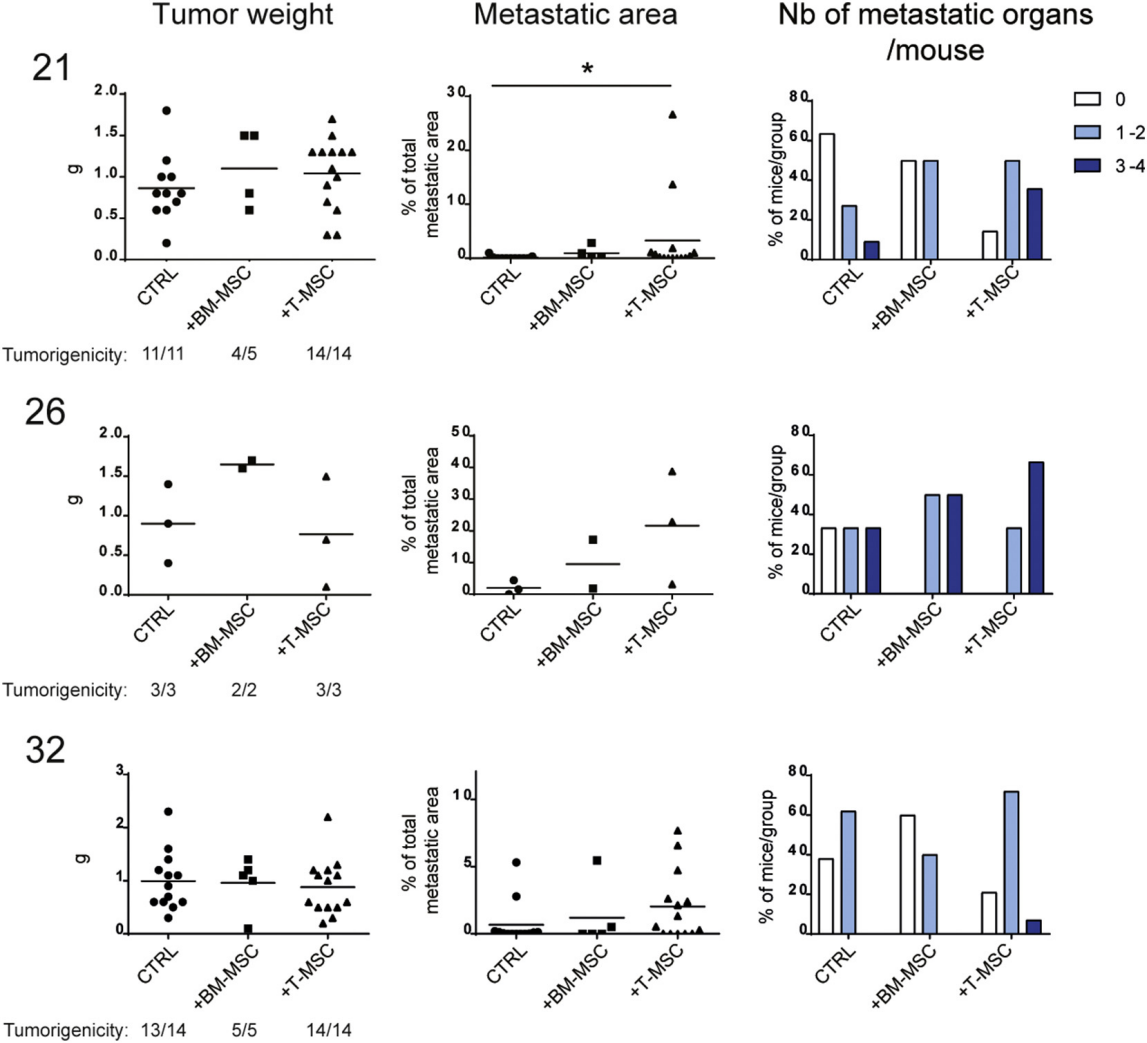
T-MSCs promote the metastatic potential of paired primary lung cancer cells more efficiently than BM-MSCs. Results from mouse injections with 21, 26 and 32 primary tumor cells alone (CTRL) or in the presence of BM-MSCs (from one healthy donor, BM1) or paired T-MSCs. For 21 and 32 tumors, 2 independent experiments were performed and data pooled together. Numbers of total injected mice per group are indicated as the denominator for the assessment of tumorigenicity. For each group, the tumorigenic ability is reported as the ratio between the number of mice with tumors to the total number of injected mice. Mice without tumors (n = 2) were not included in analyses or graphs. Tumor weights (grams, (g); left panels), percentages of total metastatic area per mouse (middle panels) and proportions of mice per group with 0 (white bars), 1–2 (clear blue) or 3–4 (dark blue) metastatic organs (right panels) are depicted for the three groups. Horizontal lines represent mean values. Groups were compared using the nonparametric Kruskal–Wallis (K–W) test with post-hoc Dunn's multiple comparison test. Significant differences are reported as *according to the level of significance (P < 0.5). See also Fig. S2.

To address the effect of primary tumor-associated MSCs on tumor progression, we compared tumor formation resulting from co-injection of tumor cells and T-MSCs to that arising from co-injection of tumor cells with unrelated BM-MSCs and injection of tumor cells alone. Although the small numbers of MSCs in the resected tumors are likely to have little impact on the global behavior of large primary tumors, their effect on small tumor cell subpopulations within the tumor mass with which they are in contact may be highly relevant. Low cell numbers at a 1:1 MSC:tumor cell ratio were therefore used in all co-injections. Tumor engraftment was comparable among the three groups for all primary samples (Fig. 2). In contrast, a significantly increased metastatic load was observed when tumor cells from sample 21 were co-injected with paired T-MSCs (P = 0.0242), with samples 26 and 32 displaying the same trend (Fig. 2). Metastases occurred mainly in liver, lung and spleen and in the contralateral kidney of mice injected with cells from sample 21 (P = 0.0098; Fig. S2B). Moreover, cells from all tumor samples co-injected with T-MSCs formed multi-organ metastases whereas their co-injection with BM-MSCs resulted in a more modest metastatic spread (Fig. 2, right panels) with the exception of the spleen, which displayed a comparable metastatic load (Fig. S2B). These observations suggest that MSCs preferentially enhance dissemination of primary tumor cells rather than their tumor forming ability at the site of injection and that although both BM- and T-MSCs promote metastasis, T-MSCs display a more potent effect.

### 3.3 T-MSCs and Paired N-MSCs Display Different Gene Expression Profiles

**Fig. 3:**
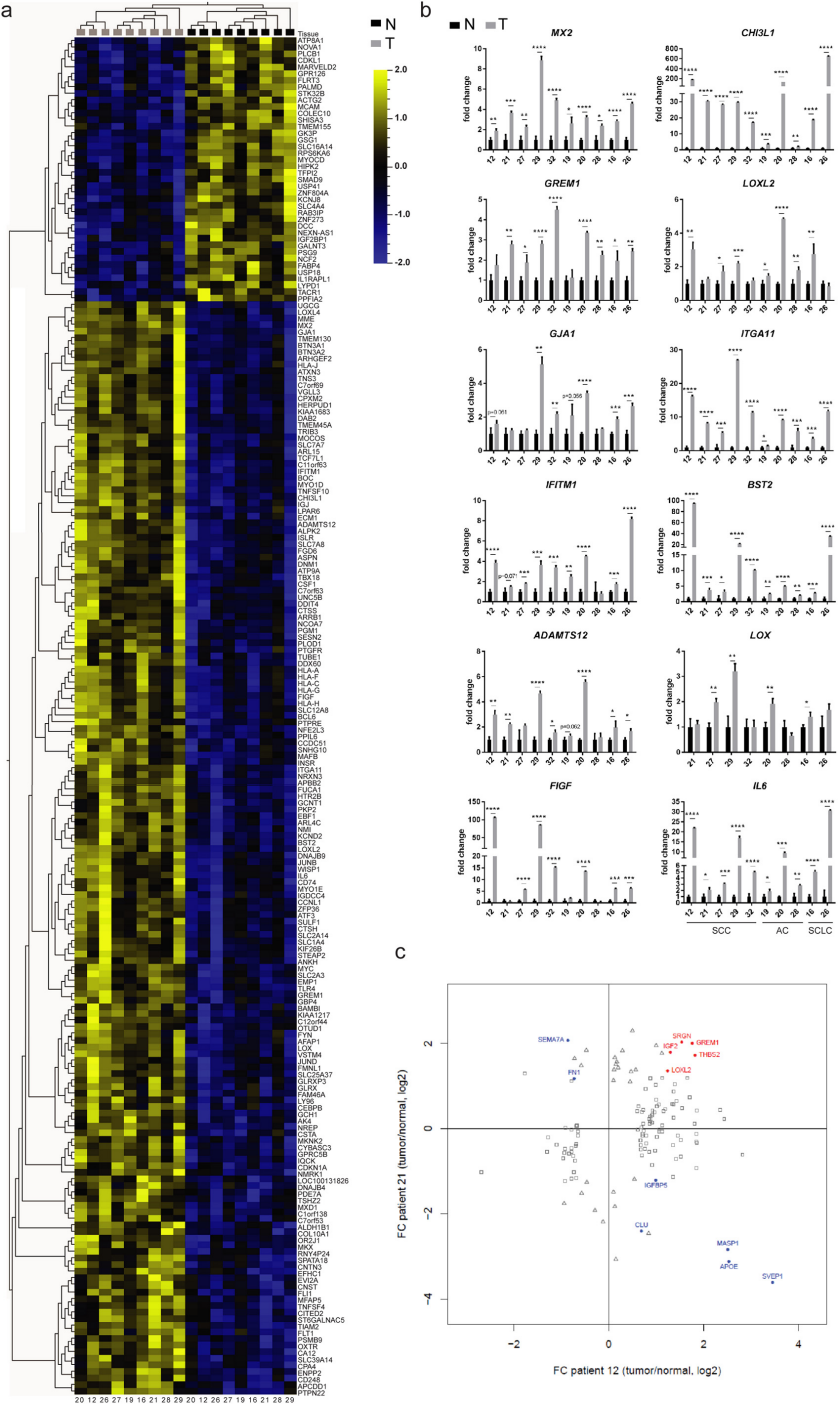
Comparison between primary samples of paired N- and T-MSCs. (A-B) Comparison between the expression profile of N- (black) and T-MSC (grey) samples isolated from the same patients. (A) Microarray heatmap showing a distinct mRNA expression profile between paired samples from 9 patients. Log2 expression values are indicated by a color scale: up-regulated genes are depicted in yellow, down-regulated genes in blue. Each column represents a MSC sample (patient identification numbers (ID) are reported at the bottom) while rows represent genes identified by symbols on the right of the heatmap. Dendrograms are based on hierarchical clustering of samples/variables. (B) Validation by quantitative Real-Time PCR of selected genes. Samples are ordered according to tumor subtype (SCC, AC or SCLC) and patient ID are indicated below each graph. For all genes and for each patient (n = 10), expression was normalized to N-MSC levels using the *PP1A* housekeeping gene. Median values and SD from triplicates are shown. Paired N- and T-MSC expression levels were compared using multiple t tests. Significant differences were indicated according to the level of significance: * at P ≤ 0.05; ** at P ≤ 0.01; *** at P ≤ 0.001; **** at P ≤ 0.0001. (C) Secretome analysis of paired N- and T-MSC samples from patients 12 and 21. In red, proteins found to be significantly up-regulated in both T-MSC samples. In blue, proteins found to have significantly different abundance in the two samples but in opposite direction. Squares represent significant differentially secreted proteins from patient 12, triangles from patient 21. See also Fig. S3 and Table S1. (For interpretation of the references to color in this figure legend, the reader is referred to the web version of this article.)

Although tumor-associated MSCs may be recruited from various sites, including the bone marrow and adipose tissues, the most likely source are normal tissue-resident MSCs, warranting phenotypic and functional comparison between paired T- and N-MSCs. Microarray analysis of 9 primary paired samples (the 10th sample was obtained at a later date) indicated that T- and N-MSCs display distinct gene expression profiles. Using a fixed variance filter 0.128 (σ/σ_max_) and a 0.2 q-value (FDR) cut-off, we identified 205 differentially expressed genes (P-value < 0.01039): 165 genes were upregulated and 40 downregulated in T-MSCs (Fig. 3A). The observed gene modulation was independent of the tumor subtype and 12 among the 50 most significantly up-regulated (P < 0.00252; fold changes 1.22 < x < 9.36; Table S1) genes were selected for validation, including: *MX2*, *CHI3L1*, *GREM1*, *LOXL2*, *GJA1*, *ITGA11*, *IFITM1*, *BST2*, *ADAMTS12*, *LOX*, *FIGF* and *IL6* (Fig. 3B). Validation was performed on 10 pulmonary T- and N-MSC sample pairs. With the exception of *GJA1* and *LOX*, which were found to be significantly more highly expressed in T-MSCs in only 5 and 4 pairs of samples respectively, increased expression in T-MSCs of all the remaining genes was validated in at least 7 out of 10 pairs (Fig. 3B). Nine of the genes selected for initial validation were used in subsequent experiments.

BM-MSCs from three unrelated adults displayed higher *LOXL2*, *GJA1* and *ITGA11* expression than most T-MSCs whereas *GREM1* and *ADAMTS12* expression was comparable in BM- and T-MSCs (Fig. S3A). *CHI3L1*, *BST2*, *IL6*, *MX2*, *IFITM1*, *LOX* and *FIGF* were more weakly expressed or undetectable in BM-MSCs, more closely resembling the N-MSCs profile (Fig. S3A). With respect to the selected panel of genes, BM-MSCs therefore displayed a phenotype that was distinct from that of T- and N-MSCs.

Because several of the selected genes encoded secreted proteins, we assessed the secretome of N- and T-MSCs from two patients with SCC (12 and 21) and of BM-MSCs from two healthy donors. Twelve proteins displayed significantly different abundance in the supernatants (SN) of MSCs from both patients (Fig. 3C), but only five (GREM1, LOXL2, SRGN, THBS2 and IGF2) were increased in the SNs from both 12 and 21 T-MSCs (in red). These five secreted proteins were also found in the conditioned media of the two batches of BM-MSCs at levels that were higher than (SRGN and THSB), comparable to (GREM1 and LOXL2) and lower than (IGF2) those observed in T-MSC supernatants (Fig. S3B). LOXL2 enrichment was validated by Western blot analysis of freshly prepared SNs from N- and T-MSCs from patients 21, 26, 32, as well as from patient 29 (Fig. S3); only patient 29 samples had comparable LOXL2 levels in N- and T-MSC SNs. Among the non-secreted proteins, assessment of ITGA11 by Western blot analysis revealed its expression to be increased in all T-MSC cell lysates compared to their N-MSC counterparts (Fig. S3).

### 3.4 The Tumor Microenvironment Induces N-MSCs to Acquire a T-MSC Expression Profile

**Fig. 4:**
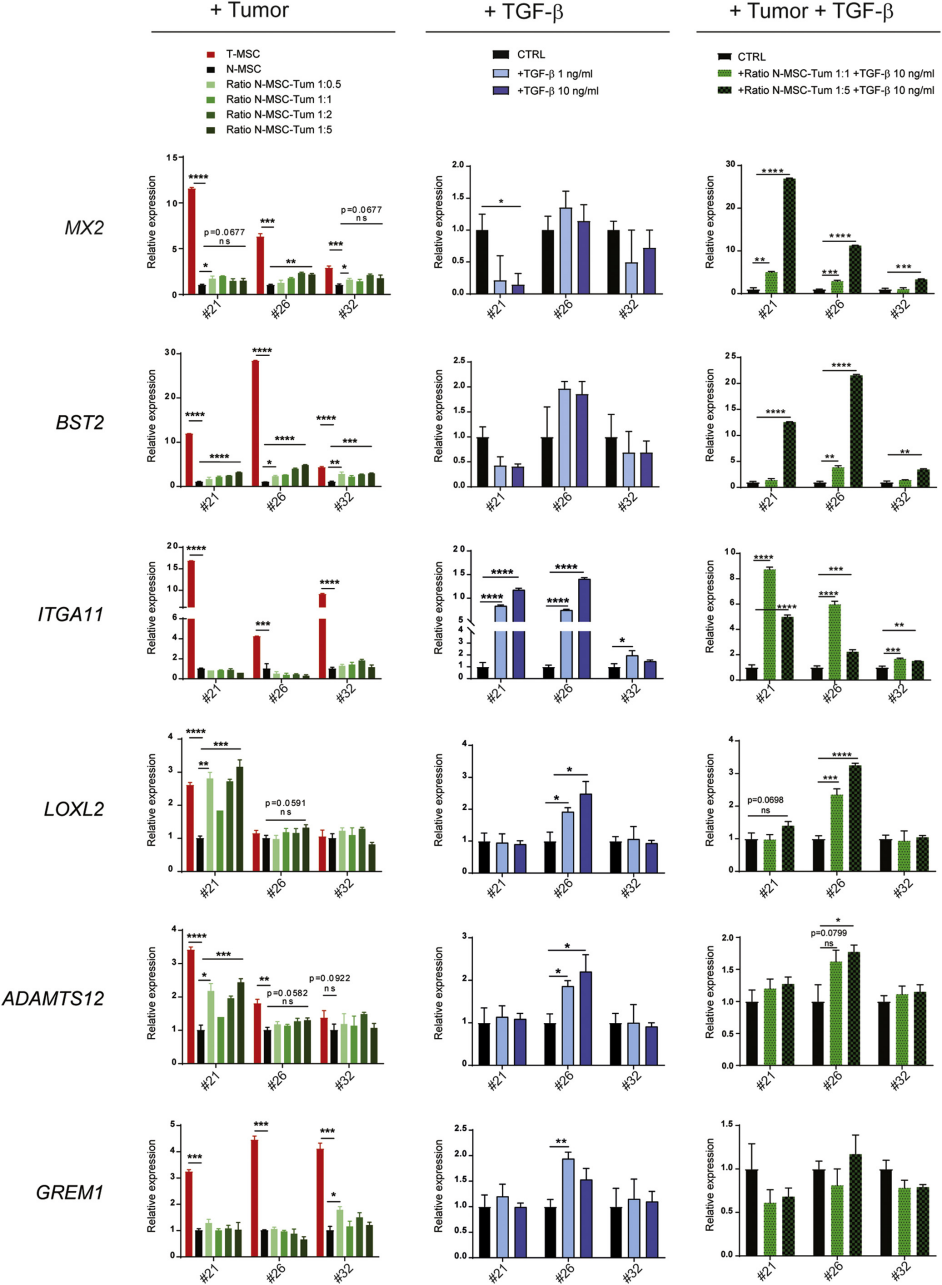
N-MSC phenotype is modulated by different components of the tumor microenvironment. (Left column) N-MSCs from patients 21, 26 and 32 were cultured for 7 days alone (black bars) or with increasing numbers (green color scale bars) of primary tumor cells from the same patient in indirect conditions (1-μm pore size inserts); N-MSC:tumor cell ratios are indicated. The indicated gene expression was assessed by qRT-PCR and normalized to N-MSCs cultured alone using the *PP1A* housekeeping gene (fold change = 1). T-MSCs alone cultured under the same conditions (red bars) are shown as a reference. Data represent median values and SD from triplicate assays. For statistical analysis, N-MSCs alone were compared with T-MSCs or with N-MSCs in presence of the lowest or the highest number of tumor cells. (Middle column) N-MSCs were treated for 7 days with TGF-β1 at 1 ng/ml (light blue bars) or 10 ng/ml (dark blue bars). Gene expression levels normalized to untreated N-MSCs (black bars) using the *TBP* housekeeping gene (fold change = 1) are indicated. Median values and SD from triplicates are shown. (Right column) N-MSCs from patients 21, 26 and 32 were cultured for 7 days alone (black bars) or with primary tumor cells from the same patient at 1:1 (light green bars with black dots) or 1:5 (dark green bars with black dots) N-MSC:tumor cell ratios and treated with TGF-β1 at 10 ng/ml. Expression of the reported genes was assessed by qRT-PCR and normalized to untreated N-MSCs cultured alone using the *TBP* housekeeping gene (fold change = 1). Median values and SD from triplicate assays are shown. For all experiments, gene expression levels were compared by multiple t tests using the Holm-Sidak correction method. The adjusted P-values from the comparisons of N-MSCs alone and/or untreated with N-MSCs cultured in other conditions are indicated according to the level of significance: * at P ≤ 0.05; ** at P ≤ 0.01; *** at P ≤ 0.001; **** at P ≤ 0.0001. When almost significant (P ≤ 0.1), P-values are also reported with the symbol “ns” (not significant). See also Fig. S4A.

To address the possibility that T-MSCs may originate from resident N-MSCs in response to tumor cell and TME-derived signals, we cultured N-MSCs from patients 21, 26 and 32 in the presence of increasing numbers of paired primary tumor cells and assessed expression of 9 validated genes at 3, 5 (not shown) and 7 days (Figs. 4 and S4A, left columns). On day 7, the expression of 5 out of the 9 genes was significantly induced in N-MSCs from at least one patient proportionally to increasing tumor:MSC cell ratios. *IL6* and *BST2* were significantly induced in all N-MSC samples already after 3 days, with *IL6* reaching levels comparable to those in T-MSCs. In contrast, *ADAMTS12*, *MX2*, *LOXL2* and *GREM1* expression varied during the course of the assay, often displaying transient induction in the different N-MSC cultures. Other genes, including *FIGF*, *ITGA11* and *CHI3L1* were not induced in any of the culture conditions. Altogether, our results show that tumor cells alone can modulate the N-MSC gene expression profile, but that the resulting changes only partially recapitulate T-MSC features. Mediators generated by components of the TME most likely contribute to the establishment of the full T-MSC phenotype.

Inflammation and tissue remodeling are inherent to the TME (Landskron et al., 2014; Shi et al., 2015), which, as a consequence, is rich in a wide range of cytokines. A key cytokine in tissue repair is transforming growth factor β (TGF-β), which orchestrates wound healing and has a dual effect on cancer, displaying both tumor suppressive and tumor enhancing properties, depending on the context and stage of tumor progression (Ikushima and Miyazono, 2010). TGF-β was expressed comparably in the tumor cells and MSCs, as assessed by qRT-PCR (Fig. S4B) but unlike N- and T-MSCs, tumor cells did not secrete detectable levels of TGF-β1 (Fig. S4B). Using Cytoscape software for the analysis of molecular interactions and pathway connectivity, we found that TGF-β1 could be potentially connected to proteins encoded by several of the genes found to be upregulated in T-MSCs, especially *IL6* (Fig. S4C). We therefore subjected N-MSCs (21, 26 and 32) to both cytokines and assessed the changes in the N-MSC phenotype at 3, 5 (data not shown) and 7 days of treatment with each. Whereas significant IL-6-mediated induction of expression of the selected genes was limited to *BST2* and *MX2* in sample 26 N-MSCs (Fig. S4D), TGF-β1 enhanced the expression of multiple genes (Figs. 4 and S4A, middle columns). *ITGA11* and *FIGF* were the most significantly up-regulated genes in 21 and 26 N-MSC samples, reaching levels comparable to those observed in T-MSCs (Figs. 4A and S4A). *GREM1*, *ADAMTS12* and *LOXL2* were upregulated only in N-MSCs from patient 26 and *IL6* only in patient 21 N-MSCs after 7 days of treatment whereas TGF-β1 had no significant enhancing effect on *BST2*, *MX2* or *CHI3L1* expression. Combined treatment using the two cytokines recapitulated the effect of TGF-β1 alone (not shown). Addition of TGF-β to tumor cell-N-MSC co-culture augmented *MX2*, *BST2* and *IL6* (Figs. 4 and S4A, right columns) but not *ADAMTS12*, *LOXL2 GREM1* or *CHI3L1* expression in N-MSCs. At a 5:1 tumor cell-N-MSC ratio, tumor cell presence appeared to attenuate *ITGA11* upregulation by TGF-β in sample 21 N-MSCs (Fig. 4, right column). Conversely, TGF-β abrogated tumor cell-co-culture-dependent augmentation of *LOXL2* and *ADAMTS12* expression in patient 21 N-MSCs (Fig. 4, right column). Thus, TGF-β modulation of the N-MSC phenotype may be affected by tumor cell presence and may in turn alter the effect of tumor cells on MSCs in a manner that cannot be readily predicted. These observations underscore the complexity of tumor cell-TME interplay in shaping host tissue stromal behavior.

### 3.5 N- and T-MSCs Promote Metastases of Paired Tumor Cells and Promote Tumor Cell Dissemination in 3D Gels

**Fig. 5:**
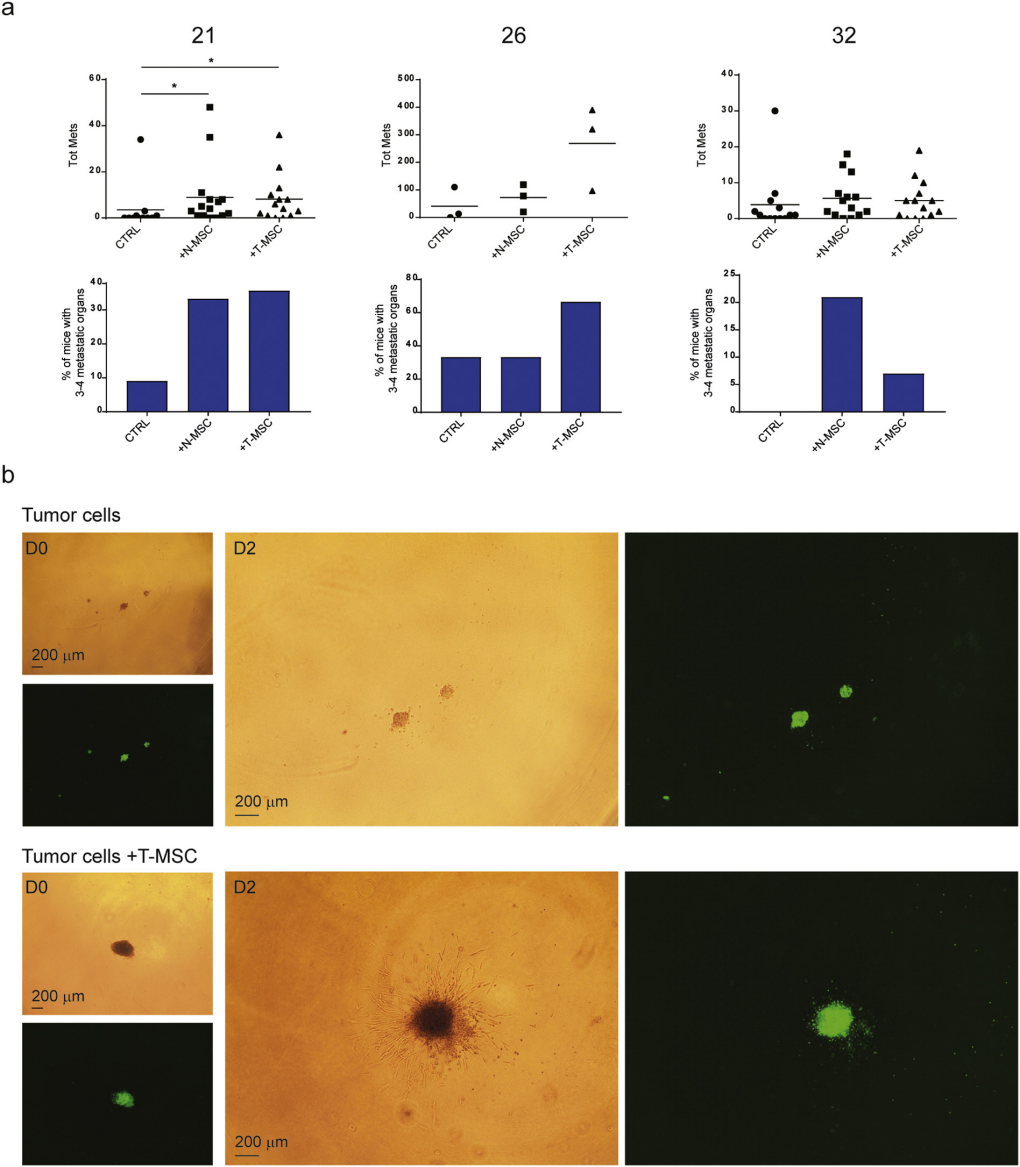
*In vivo* effects of N- and T-MSCs on primary lung cancer cells and MSC:tumor cell interactions in three dimensional structures. (A) Results from mouse injections with 21, 26 and 32 primary cancer cells alone (CTRL; n = 11, 3 and 13 mice respectively) or in presence of paired N- (n = 15, 3, 14 mice respectively) and T-MSCs (n = 14, 3, 14 mice respectively). For 21 and 32 tumors, 2 independent experiments were performed and data pooled together. Top panels: For each group of injection, total numbers of metastases per injected mouse are reported. Means are depicted by horizontal lines. Groups were compared using the nonparametric Kruskal-Wallis (K-W) test with post-hoc Dunn's multiple comparison test. Significant differences are reported and adjusted P-values indicated as * at P < 0.05. Lower panels: percentages of mice bearing metastases in 3–4 organs simultaneously in each group. See also Fig. S5. (B) Spheroids of CFSE-labeled tumor cells alone or mixed with T-MSCs from patient 21 at a 1:1 tumor:MSC cell ratio included in a 3-D matrix. Cell dissemination and three dimensional cell interactions were monitored by microscopy and the experiment performed in quadruplicate. One representative spheroid of tumor cells alone or mixed with T-MSCs is shown at day 0 and day 2 and images taken at 4× magnification by light and fluorescent microscopy. Scale bar = 200 μm. See also Fig. S6.

Based on our observations that primary tumor cells can modulate the phenotype of N-MSCs *in vitro*, it would seem reasonable to expect mutual modulation of behavior between MSCs and tumor cells. N-MSCs « educated » by the tumor cells and the TME to become T-MSCs may thus signal back to the tumor to support progression of some of its cell subpopulations. We therefore co-injected 21, 26 and 32 primary tumor cells with N-MSCs from the same patients and assessed tumor growth and dissemination. Whereas we did not observe any difference in tumor weight at the site of injection (data not shown) the two SCC tumor samples (21 and 32) co-injected with N-MSCs displayed higher metastatic activity, similar to that resulting from co-injection with T-MSCs (Fig. 2, Fig. 5A and S5B). The number of mice bearing metastases in multiple organs increased in both 21 and 32 tumor cell co-injections with N-MSCs compared to injection of tumor cells alone. In fact, 32 N-MSCs induced higher metastatic activity of the paired tumor cells than the corresponding T-MSCs (Fig. 5A). The total number of metastases correlated with metastatic tumor size (Fig. S5A). N-MSCs promoted tumor 21 and 32 metastases to the liver and spleen, as well as the kidney for tumor 21 (Fig. S5B). In contrast, co-injection of the composite carcinoma 26 tumor cells with paired N-MSCs failed to enhance their metastatic activity (Figs. 5A and S5B). The influence of tumor cells in N-MSC transition to the T-MSC phenotype may therefore vary according to tumor cell properties. In some cases, the T-MSC phenotype may be primarily sculpted by the TME, which may display at least some common features among different tumor types. Consistent with this notion SCC- and composite tumor-derived MSCs had a similar transcriptome. It is also noteworthy that T-MSCs maintain their phenotype in culture, at least for the number of passages to which they were subjected in the present study, indicating that the phenotype is stable, and not dependent on continued tumor cell presence.

To determine whether MSCs may directly influence tumor cell migration, we assessed tumor cell detachment from spheroids in the presence and absence of MSCs in 3D gel culture. Tumor spheroid co-culture with T-MSCs in 3D gels revealed progressive individual tumor cell detachment from spheroids along the path of MSC spreading (Figs. 5B and S6), suggesting that MSCs may physically facilitate tumor cell dissemination. Spheroid cultures in the absence of MSCs remained compact without significant cell detachment (Figs. 5B and S6). One mechanism by which MSC may promote tumor dissemination may therefore be the creation of a path for tumor cells to travel along, possibly led by migrating MSCs.

### 3.6 N- and T-MSCs Induce Similar Gene Expression Changes in Tumor Cells

**Fig. 6:**
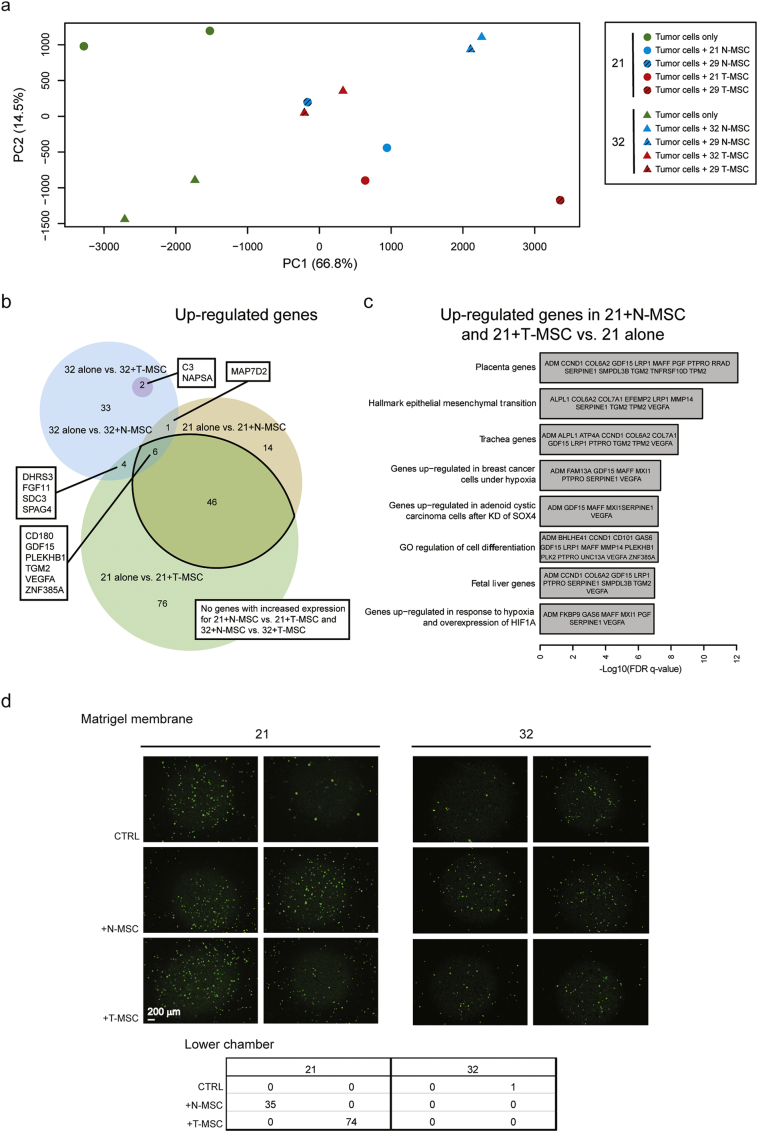
Effects of N- and T-MSCs on primary lung cancer cell transcriptome and invasiveness. (A–C) Analysis of RNA-Seq data from primary tumor cells cultured alone or in the presence of N- or T-MSCs from the same patient or from patient 29. Two independent experiments were performed with paired or 29 MSCs. (A) Principal component analysis (PCA) plot of RNA-Seq data from 21 (circles) and 32 (triangles) tumor cells cultured alone (in green) or with N- (in blue) or T-MSCs (in red) isolated from the same patient (filled symbols) or from patient 29 (striped) after removal of sample and batch effects. Percentages of data variation explained by the two first principal components (PC) are reported in brackets. (B) Venn diagram of genes up-regulated by tumor cells after MSC co-culture. For the analysis, results from co-cultures with MSCs (N- or T-) from different patients (paired or 29) were pooled together and indicated as “+N-MSC” or “+T-MSC”. Genes up-regulated in 32 tumor cells cultured with N-MSCs are represented by a blue circle, containing a smaller darker one that represents the 2 overlapping genes also overexpressed following T-MSC co-culture. Genes up-regulated in tumor 21 in the presence of N- or T-MSCs are respectively symbolized by a yellow and a green intersecting circles. The size of circles represents the numbers of up-regulated genes for each culture condition. The number of genes included in each region is indicated. Overlapping gene expression in the different conditions is listed (rectangles). (C) Gene ontology analysis of up-regulated genes in 21 tumor cells co-cultured with MSCs. See also Table S2. (D) Invasiveness of CFSE-labeled tumor cells (upper chamber, medium without serum) from patients 21 and 32 through a matrigel membrane was assessed in transwell co-culture conditions with N- or T-MSCs (lower chamber, medium with serum). Medium with serum in the absence of MSCs in the lower chamber was used as the control condition (CTRL). Experiments in each condition were performed in duplicate. Following overnight incubation, images of CFSE-labeled tumor cells invading the matrigel were captured at the level of the transwell membrane after removal of non-invading cells from the upper chamber by washing the gel surface multiple times with PBS. Images were taken by fluorescent microscopy (with the focus adjusted to the 8 μm insert pores by light microscopy) at 4× magnification. Scale bar = 200 μm. The table indicates the numbers of CFSE-labeled tumor cells in the lower chamber at the end of the co-culture. Cells were counted on images taken by fluorescent microscopy at 4× magnification in the lower chamber, with the focus adjusted to the MSCs by light microscopy.

To further explore mechanisms by which MSCs may enhance dissemination of paired tumor cells, we addressed, by RNA-Seq, the changes in the gene expression profile of tumor cells induced by co-culture with lung MSCs. Based on their comparable histology and their similar behavior *in vivo*, we focused on samples 21 and 32. To address the general applicability of the effect of MSCs on SCC progression, we also co-cultured the tumor cells with unrelated T- and N-MSCs derived from patient 29. The PCA plot of RNA-Seq data showed similar results when each of the tumor cell batches was cultured with paired or sample 29 MSCs. The similarity was particularly marked when sample 32 tumor cells were used (Fig. 6A). Thus, in subsequent analyses they were considered as replicates and data were pooled together. Principal component 1 (PC1) revealed that tumor cells cultured alone (green symbols) display a markedly distinct transcriptome from the same cells following indirect co-culture with MSCs (blue and red symbols; Fig. 6A). In contrast and mirroring the results obtained from *in vivo* experiments, variation between tumor cell transcriptomes following co-culture with N- or T-MSCs was far more limited, precluding differential clustering of samples according to the two co-culture conditions. As a result, we observed a highly significant overlap among genes modulated (P-value < 0.05, fold change > 2 and detectable expression) in tumor cells by exposure to N- or T-MSCs (Fig. 6B and Table S2), consistent with the observation that tumors contribute to N-MSC acquisition of a T-MSC phenotype. Substantial variation in terms of gene expression changes was observed between the two tumors upon exposure to MSCs (Fig. 6B and Table S2), possibly explaining the differences in metastatic number and volume induced by MSCs (Fig. 5A). Similarly, the number of genes modulated by T- and N-MSCs differed and in 32 tumor cells, more genes were modulated by N- than by T-MSCs (Fig. 6B and Table S2). As expected, some genes were modulated in both 21 and 32 tumors following N- or T-MSC co-culture. Thus, *KALRN* was downregulated in all settings, whereas 6 upregulated genes were shared among 21 + N-MSC, 21 + T-MSC and 32 + N-MSC co-cultures, including *CD180*, *GDF15*, *PLEKHB1*, *TGM2*, *VEGFA* and *ZNF385A*. Some of these genes are reported to be associated with epithelial-mesenchymal-transition (EMT) and hypoxia, both of which are implicated in metastasis (Gilkes et al., 2014; Lu and Kang, 2010; Tsai and Yang, 2013). Gene ontology (GO) analysis of the 52 genes upregulated in 21 tumor cells following both N- and T-MSC co-culture further showed significant enrichment in genes related to EMT and hypoxia (Fig. 6C). Interestingly, the most significantly overrepresented GO term included genes related to the placenta, recently reported to be associated with aggressive, metastasis-prone lung cancer (Rousseaux et al., 2013). Altogether, our co-culture experiments indicate that primary MSCs can modulate the expression profile of paired lung cancer cells, inducing genes that are associated with an aggressive tumor phenotype and possibly explaining their increased metastatic potential *in vivo*.

To determine whether the observed changes in the tumor cell transcriptome may correlate with enhanced invasiveness, we assessed patient 21 and 32 tumor cell behavior in transwell assays in the presence and absence of N- and T-MSCs. MSCs were cultured in the bottom chamber in the presence of serum, whereas CFSE-labeled tumor cells were seeded onto matrigel in the top chamber in the absence of serum. Tumor cell migration was scored following overnight incubation after removal of cells that had remained on the surface of the gel. Fluorescent cells were counted in the lower chamber (Fig. 6D, lower panel) and were also visualized on the transwell membrane (Fig. 6D, upper panel). Both N- and T-MSCs promoted invasion of the gel and penetration into the lower chamber of sample 21 tumor cells (Fig. 6D, lower panel). Cells from tumor 32 displayed enhanced entry into the matrigel in response to N-MSCs, as assessed by fluorescence on the transwell membrane (Fig. 6D) but did not penetrate the lower chamber within the experimental time frame.

### 3.7 *GREM1*, *LOXL2*, *ADAMTS12* and *ITGA11* Expression by MSCs Promote Tumor Cell Metastasis

To further investigate the mechanism by which MSCs promote tumor dissemination, we sought to identify genes whose expression was induced in MSCs by cancer cells or the TME that may be functionally implicated in promoting lung cancer metastasis. Among the genes that were upregulated in T-MSCs, we selected *GREM1* and *LOXL2*, whose increased expression was validated by qRT-PCR and whose encoded proteins were significantly enriched in T-MSC supernatants (Fig. 3). Interestingly, analysis of numerous gene expression datasets from tumor samples of non-small cell lung carcinoma patients available in the R2 genomics database, revealed that expression of these two genes is highly correlated. Remarkably, in all tested microarrays, *GREM1* and *LOXL2* were associated with the expression of two other genes among the top 30 genes upregulated in T-MSCs (Table S1), *ADAMTS12* and *ITGA11*, whose increased expression we had already validated. To further probe the putative relationship between these four genes, we examined a microarray heatmap of genes whose expression is strongly correlated with *GREM1* from one representative dataset, “Tumor Non-small cell lung carcinoma - Plamadeala - 410 - MAS5.0 - u133p2” (Huang et al., 2015) (Fig. S7A). The dataset does not distinguish between gene expression in tumor cells and in the stroma. However, in our samples, the four genes were expressed in T-MSCs, and more modestly in N-MSCs but not (*GREM1* and *LOXL2*) or weakly so (*ITGA11* and *ADAMTS12*) in the tumor cells (data not shown). They were also highly expressed in BM-MSCs, which promoted metastasis to the spleen but less so to other organs. Assessment of the correlation of the 4-gene expression in other data sets revealed significant relatedness between *GREM1*, *ADAMTS12* and *LOXL2* expression in NSCLC-associated MSCs (Table S3). However, the same correlation was not found in NSCLC-associated CAFs, the only significant relatedness in the latter being observed between *ADAMTS12* and *ITGA11* expression (Table S3). Because of their strong co-expression and their reported roles in ECM and tissue remodeling, we addressed the possibility that *GREM1*, *LOXL2*, *ADAMTS12* and *ITGA11* may constitute a candidate core pro-metastatic MSC gene signature. As our primary tumor model, we used sample 26 tumor cells, which displayed a marked difference in metastasis formation upon exposure to N- or T-MSCs (Fig. 5A).

**Fig. 7:**
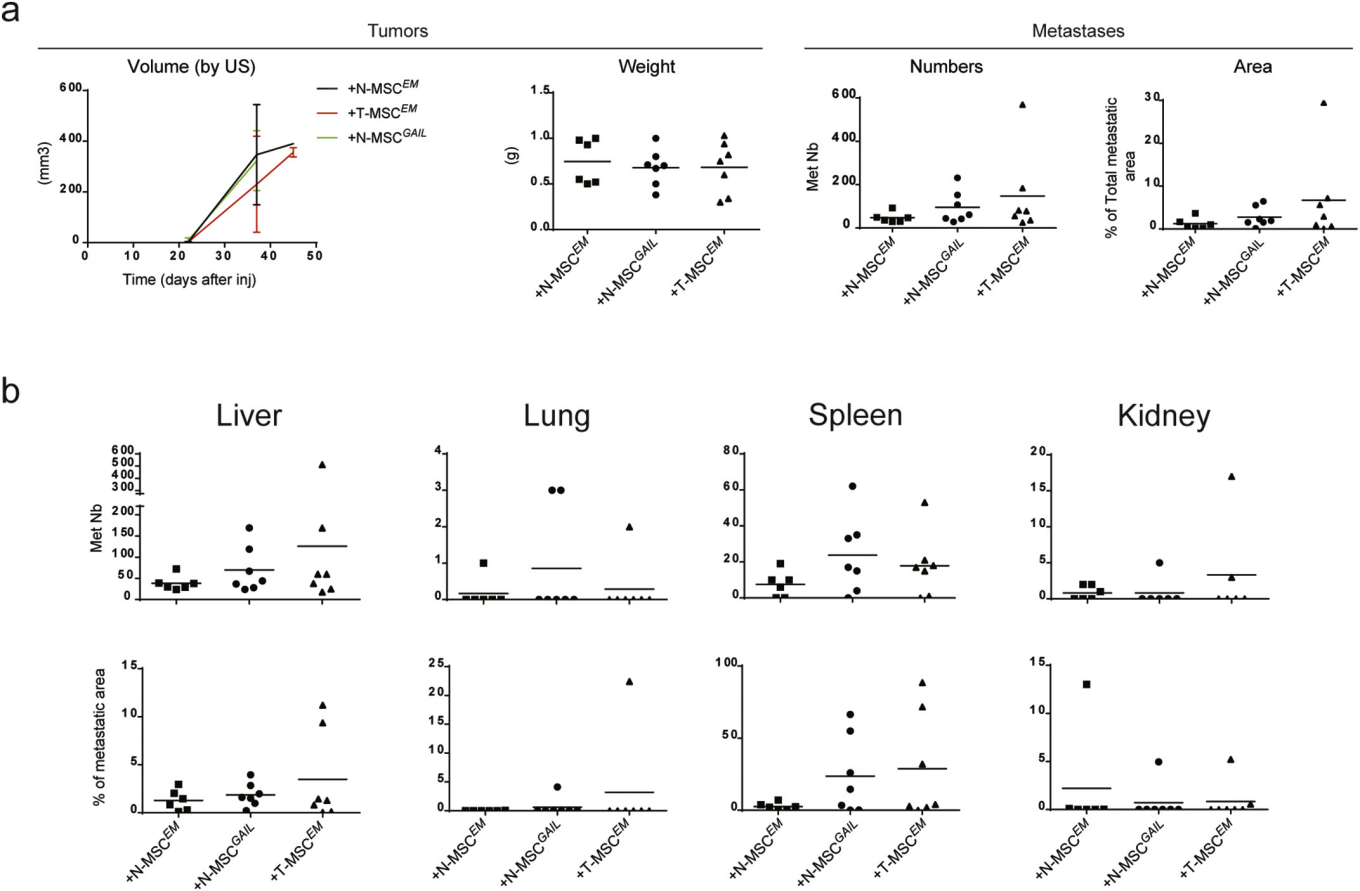
N-MSCs overexpressing *GREM1*, *LOXL2*, *ITGA11* and *ADAMTS12* increase the metastatic potential of paired tumor cells. Results from 26 tumor cell co-injections with paired N- (n = 6) or T-MSCs (n = 7) expressing the *Emerald* reporter gene (*EM*) or with a bulk of N-MSCs single expressing *GREM1*, *LOXL2*, *ITGA11* and *ADAMTS12* (*GAIL*; n = 7) in addition to the *EM* gene. (A) Left: ultrasound (US) follow-up of tumor volume (mm^3^) from the day of cell injection to sacrifice is shown. Lines connect the mean values for each group of mice. Standard deviations are indicated. Right: for each group, the tumor weight (g) quantification of the total number of metastases and proportion of the total tissue area occupied my metastases per mouse are shown. Horizontal lines indicate mean values. (B) Number of metastases and the proportion of each affected organ occupied by metastatic tumor growth per mouse in the three groups of mice are shown. Mean values are indicated by the horizontal lines. Groups were compared using the nonparametric Kruskal–Wallis (K–W) test with post-hoc Dunn's multiple comparison test. See also Fig. S6.

We first infected 26 N-MSCs with either Emerald control virus (N-MSC^*EM*^) or with single-gene containing viruses at a low MOI to recapitulate the increase in the corresponding gene expression observed in T-MSCs (Figs. 3B and S7B). 26 T-MSCs infected with EM control virus (T-MSC^*EM*^) were used as an additional control. As suggested by the dataset analysis reported above, expression of the four genes appeared to be linked as introduction of one gene increased the endogenous expression levels of the others, with the exception of *ADAMTS12*. However, introduction of *ADAMTS12* augmented expression of the three other genes (Fig. S7B). To overcome the difficulty of expressing multiple exogenous genes in primary MSCs, we engineered MSCs that express each of the four genes individually and equal numbers of the corresponding MSCs were pooled to generate a bulk of MSCs expressing the four genes (MSC^*GAIL*^). The MSC bulk was then co-injected with sample 26 tumor cells (2000 cells per mouse) at a 1:1 MSC^*GAIL*^:tumor cell ratio and compared with tumor cells injected with T-MSC^*EM*^ or N-MSC^*EM*^. Similar to our initial tumor cell/paired MSC co-injections (Fig. 2), we did not observe any difference in local tumor growth among the three conditions (Fig. 7A). In contrast and similar to our observations using non-infected MSCs, 26 tumor cells co-injected with T-MSC^*EM*^ displayed higher metastatic activity than those co-injected with N-MSC^*EM*^ (Fig. 7A and B). Interestingly, following infection with the EM control virus, T-MSCs displayed increased expression only of *GREM1* and *ITGA11* compared to N-MSC^*EM*^, thereby displaying a slightly different profile than non-infected T-MSCs (Fig. 3B and S7B) and possibly explaining the smaller difference in metastasis promotion between T- and N-MSC^*EM*^ than between the corresponding uninfected MSCs. Remarkably, N-MSC^*GAIL*^ clearly increased metastasis of tumor cells compared to N-MSC^*EM*^. Both the number (Nb) and the area (A) of the metastases were enhanced by N-MSC^*GAIL*^ (means: Nb = 95; A = 2.77%), to a degree that was between that of N- (means: Nb = 47.1; A = 1.21%) and T-MSC^*EM*^ (means: Nb = 147; A = 6.7%) (Fig. 7A). Metastasis quantification in each organ showed that N-MSC^*GAIL*^ promoted tumor metastases primarily in liver, lung and spleen (Fig. 7B). Altogether our data suggest that expression of *GREM1*, *LOXL2*, *ITGA11* and *ADAMTS12* by MSCs enhances primary lung cancer metastasis. Consistent with these observations, high expression of these four genes (especially *LOXL2*) in primary tumors is associated with poor overall survival in patients with lung carcinoma (Fig. S7C). Furthermore, patients with high expression of the four genes have worse prognosis than patients with elevated levels of single *GREM1*, *ADAMTS12* or *ITGA11* genes (Fig. S7D). Our data suggest that these genes constitute a stromal pro-metastatic signature in MSCs whose pharmacologic neutralization as a means to blunt lung carcinoma metastasis will be worth exploring.

## 4 Discussion

Using primary lung carcinomas and corresponding tumor-associated as well as non-tumoral adjacent tissue-derived MSCs, we have demonstrated the ability of MSCs to selectively enhance tumor metastasis without significantly affecting local growth. As our experiments were performed in NSG mice, the effects of MSCs were unrelated to their immunosuppressive functions on T and NK cells (Aggarwal and Pittenger, 2005; Galland et al., 2017; Uccelli et al., 2008). Instead, T-MSCs most likely promoted metastasis by mechanisms related to functions induced by tumor cells and the TME.

Most studies on the role of MSCs in cancer progression have been done using BM-MSCs and established tumor cell lines, both of which have limitations with respect to their representation of the physiological setting. Despite sharing the same cell surface markers and similar plasticity, BM- and T-MSCs display different features (McLean et al., 2011). Although BM-MSCs are recruited to inflammatory sites and may contribute to the TME, it seems likely that T-MSCs are primarily derived from resident N-MSCs in tissues within which the tumor develops. Consistent with this notion and unlike N-MSCs, BM-MSCs did not display the same metastasis promoting ability toward SCCs as T-MSCs, suggesting that tumor cells do not cause BM-MSCs to acquire all of the features displayed by T-MSCs. However, recent observations from our group indicate that T- and BM-MSCs display comparable immunosuppressive activity, albeit by distinct mechanisms (Galland et al., 2017), which further underscores the difference in their effect on metastasis by mechanisms unrelated to the immune response. Tumor cell lines, on the other hand, may not recapitulate the heterogeneity of primary tumor cultures in which cell subpopulations may display diverse responses to stromal cell-derived signals, only a few being endowed with metastatic potential (Naxerova et al., 2017; Ramaswamy et al., 2003). Accordingly, by secreting CXCL12 and IGF-1, mesenchymal stromal cells from triple negative breast tumors have recently been shown to positively select cancer clones with high Src activity and propensity for bone metastasis (Zhang et al., 2013).

To explore stromal-tumor cell crosstalk that is biologically and clinically relevant to human cancer progression we addressed the effects of MSCs isolated from paired primary lung carcinoma samples (T-MSCs) and normal adjacent tissues (N-MSCs) on the corresponding patient-derived tumor cells and vice-versa. Tumor-associated MSCs displayed a transcriptome distinct from that of N-MSCs, consistent with observations by others (Gottschling et al., 2013) and with the notion that tumor cells and the TME condition the MSC phenotype and function (Shi et al., 2017). Accordingly, co-culture with tumor cells resulted in N-MSC up-regulation of several of the genes that characterized the T-MSC transcriptome. Furthermore, exposure of N-MSCs to TGF-β, which orchestrates repair- and cancer-associated tissue remodeling and plays an important role in cancer progression (Ikushima and Miyazono, 2010), resulted in the induction of several genes expressed in T-MSCs that were not induced by the tumor cells. These observations suggest complementarity between the effects of tumor cells and those of the TME in promoting N-MSC transition toward a T-MSC phenotype, consistent with the participation of both tumor cells and the TME in shaping T-MSCs. It is noteworthy that several genes, which were highly expressed in T-MSCs were induced neither by tumor cells nor TGF-β in N-MSCs, suggesting the implication of other mediators from the TME. Normal tissue-associated MSCs can therefore transition toward a T-MSC phenotype in response to tumor cell and TME queues.

The most striking effect of T- and N-MSCs on primary lung carcinoma cells was the selective promotion metastasis with little or no effect on tumor growth at the site of injection. This was also the case for BM-MSCs, in albeit more limited manner and was in stark contrast to the common observation that stromal cells, often termed CAFs, promote local tumor growth first and foremost. Human T-MSC implication in the progression of other tumors, including ovarian carcinoma (McLean et al., 2011), breast cancer (Yan et al., 2012) and hepatocellular carcinoma (Yan et al., 2013) has been documented (Sun et al., 2014). However, to the best of our knowledge, the almost exclusive promotion of primary lung carcinoma cell dissemination by MSCs has not been reported before (Liu et al., 2014). Mesenchymal stem cells may acquire different properties, probably with different kinetics, according to the tumor cell type and the corresponding TME with which they are associated. Thus, N-MSCs from a patient with a composite tumor bearing predominant SCLC features were unable to promote metastasis of paired primary tumor cells, raising the possibility that the TME rather than the tumor cells played a dominant role in inducing the T-MSC phenotype in this case. However, the paucity of samples in our study precludes any generalization on tumor-type-dependent effects.

Tumor-associated MSCs modulated the primary lung carcinoma cell transcriptome, causing upregulation of placenta genes, hypoxia-induced transcripts and epithelial-mesenchymal-transition (EMT)-associated genes, all of which are reported to be related to tumor cell dissemination (Gilkes et al., 2014; Rousseaux et al., 2013; Tsai and Yang, 2013). Some of these genes, including *GDF15*, *TGM2*, *VEGFA* and *ZNF385A* were also up-regulated in primary tumor cells from patient 32 after co-culture with N-MSCs. *GDF15* expression is reported to be associated with metastasis (Aw Yong et al., 2014; Li et al., 2016) and *TGM2* (Agnihotri et al., 2013; H.-J. Kim et al., 2012) and *VEGFA* (Geng et al., 2015; Mak et al., 2010) are implicated in EMT and the response to hypoxia, respectively. High expression of VEGFA and ZNF385A correlate with poor survival of patients with serous ovarian carcinoma (Elgaaen et al., 2012). Thus, lung cancer MSCs appear to induce a gene signature associated with aggressive tumor behavior, possibly explaining, at least in part, the observed increase in metastasis.

Modulation of gene expression in both tumor cells and N-MSCs occurred rapidly *in vitro* and was for the most part due to secreted factors, as co-cultures were indirect. These observations support the largely comparable effects of SCC-derived T- and N-MSCs on tumor spread observed in our *in vivo* experiments. The difference in gene expression changes between 21 and 32 primary tumor cells in response to MSCs, which correlated with the degree of metastasis induction *in vivo*, may reflect intrinsic tumor properties, as comparable gene modulation was induced in each tumor by co-culture with either paired or allogeneic MSCs (from patient 29). But how do the ratios of MSCs to tumor cells used in our co-culture and co-injection experiments reflect the *in vivo* situation in which the MSCs populations represents no more than 4% of the tumor bulk and frequently much less? And can the small numbers of MSCs observed in the tumor bulk have a significant functional impact on tumor progression? One view is that the influence of MSCs may vary over time and as a function of tumor heterogeneity. Early in tumor development, when the tumor consists of a small cluster of cells, the ratio of tumor cells to MSCs may be comparable, in which case MSCs may have a significant influence on tumor cell behavior and promote early metastasis (Massagué and Obenauf, 2016; Wan et al., 2013). However, even at later stages, the small numbers of MSCs may influence individual tumor cells or tumor cell subpopulations. Depending on the properties and responsiveness of the latter, the effect of MSCs may lead to detachment and dissemination of individual cells or cell cohorts. Thus, even small numbers of MSCs may promote tumor progression.

An obvious question is by what mechanisms do MSCs promote tumor dissemination? In response to tumor cells and the TME, MSCs acquire the T-MSC phenotype, which may directly affect tumor cells but may also create an environment that facilitates tumor spread. By assessing the gene expression profile of T-MSCs and correlating individual gene expression with lung cancer evolution, we identified four genes that appear to be co-expressed in lung cancer patients with poor prognosis. The four genes, *GREM1*, *LOXL2*, *ADAMTS12* and *ITGA11*, provide a candidate stromal signature that may be functionally implicated in lung cancer dissemination. These four genes were also highly expressed in BM-MSCs, which although less potent in promoting multi-organ metastasis than T-MSCs, induced metastasis to the spleen at a rate comparable to that of T-MSCs. They may therefore constitute a core MSC pro-metastatic gene signature, whose potency may be modulated by the induction of other genes, possibly explaining the difference in metastasis promotion observed between T- and BM-MSCs. Consistent with this notion, expression of the four genes increased the ability of N-MSCs to promote metastasis of tumor 26, without quite reaching that of the corresponding T-MSCs. Each of the four genes is suggested to participate in tumor progression. *GREM1*, primarily expressed by stromal cells (Sneddon et al., 2006), is a bone morphogenetic protein (BMP) antagonist implicated in tumor progression (Minsoo Kim et al., 2012b) and EMT (Karagiannis et al., 2015). *LOXL2*, a member of the lysyl oxidase (LOX) family that encodes genes for copper-dependent amine oxidases, which participate in ECM stabilization by covalent cross-linking of collagen is strongly associated with cancer progression (Wu and Zhu, 2015). A pro-metastatic function of LOXL2 in murine lung cancer cells that have undergone EMT has been attributed to its role in collagen stabilization and activation of integrin signaling (Peng et al., 2017). *ITGA11*, which together with integrin β1 forms a cell surface collagen receptor, is involved in cell migration and collagen reorganization (Tiger et al., 2001). High *ITGA11* expression in the tumor stroma is a marker of poor prognosis in NSCLC patients (Chong et al., 2006; Zhu et al., 2007). *ADAMTS12* is a member of the disintegrin and metalloproteinase with thrombospondin repeats gene family reported to be endowed with a tumor protective role in several studies (El Hour et al., 2010; Fontanil et al., 2014; Wang et al., 2011). However, exogenous expression of *ADAMTS12* confers an invasive phenotype on trophoblastic cells through the induction of cell-matrix interactions by a mechanism independent of its endogenous proteolytic activity, which includes integrin up-regulation (Beristain et al., 2011). Expression of *ADAMTS12* in N-MSCs increased the expression of *ITGA11* to a level comparable to that observed in T-MSCs. Altogether, the known functions of the products of the four genes provide a plausible mechanistic explanation for MSC promotion of metastasis, which includes paracrine signaling that may maintain cancer cell pluripotency and stimulate motility as well as ECM modification that may facilitate migration. Their strong association in lung carcinoma, suggesting shared regulatory mechanisms, supports synergy among their effects, which in turn may explain the correlation of their expression with poor patient prognosis.

Most gene expression studies in cancer metastasis have focused on tumor cells in the hope of identifying metastasis-specific gene signatures or gene signatures that predict dissemination to a particular organ. However, stromal gene expression signatures that facilitate metastasis may be equally important particularly given that they arise in genetically stable cells, which may be more readily amenable to therapeutic targeting than genetically labile cancer cells. The present study, based exclusively on primary stromal and tumor cells, shows mutual transcriptome modulation between tumor cells and MSCs and uncovers a tumor cell and TME-induced stromal cell gene signature that promotes lung cancer metastasis. These observations will help provide insight into how the TME conditions cancer cell behavior and possibly open unsuspected therapeutic avenues.

## Funding Sources

This work was supported by National Science Foundation grants 310030_150024 and 310030_169563 (I.S.)

## Conflicts of Interest

The authors declare no competing financial interests.

## Author Contributions

Conceptualization: GF and IS; Investigation: GF, JV, SG, and PM; Data Analysis: GF, MQ, EM, PP; Methodology: GF, EM, CF; Resources: IL, CR and IS; Writing, Review & Editing: GF, NR and IS; Funding Acquisition: IS.

## Appendix A Supplementary data

Supplementary material Image 1
