# Supplementary material for: Reciprocal modulation of mesenchymal stem cells and tumor cells promotes lung cancer metastasis
Source: eBioMedicine. 2018 Feb 23;29:128–45. doi: 10.1016/j.ebiom.2018.02.017 (PMC5925622; doi:10.1016/j.ebiom.2018.02.017)
Supplement: Supplementary file 1 — Supplementary material [file mmc1.pdf]

Supplemental Information

Supplemental Figures, Titles and Legends

Figure S1

a

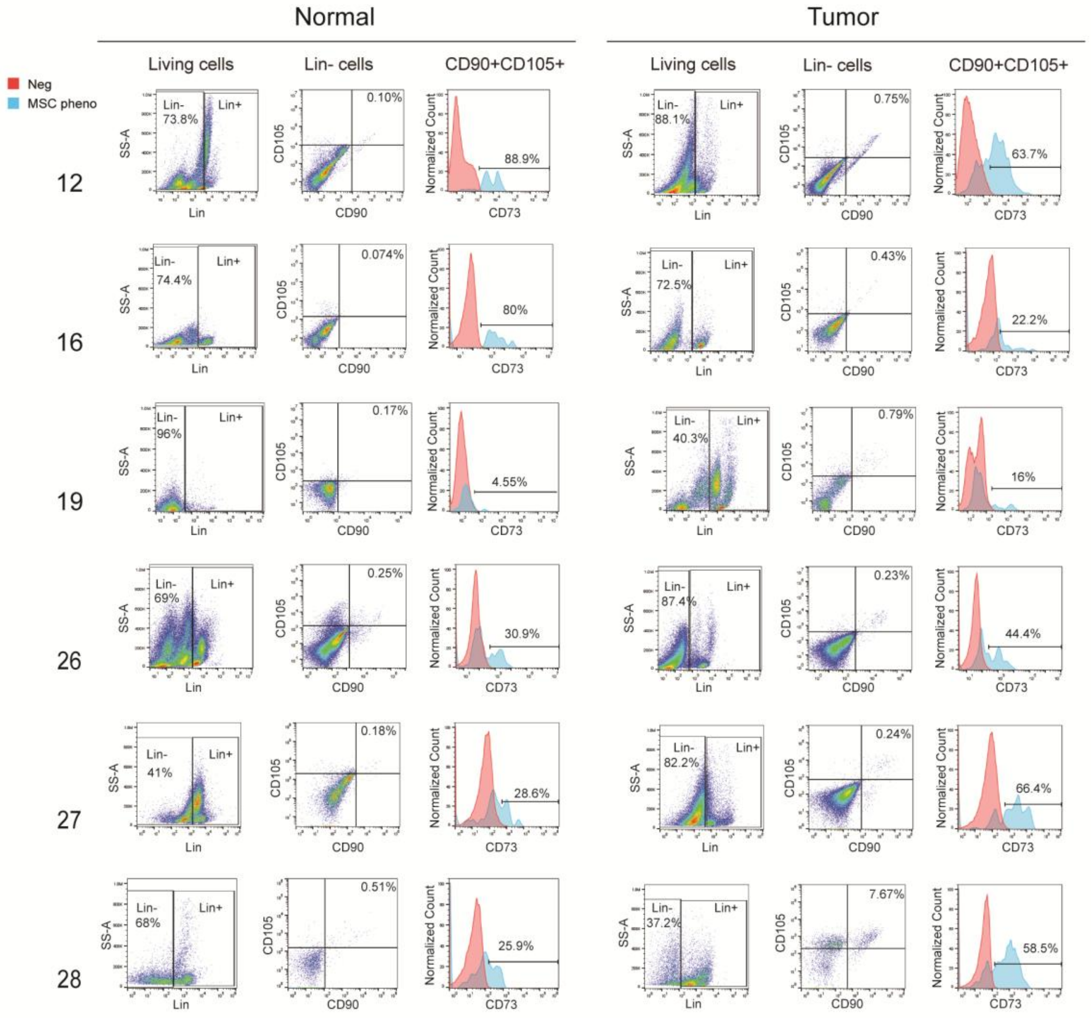

b

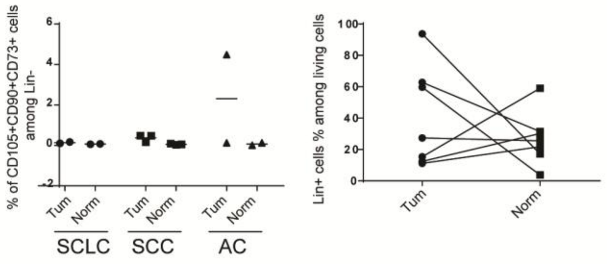

c

Healthy Donor	Gender	Age
BM1	F	51
BM2	F	54
BM3	M	78

d

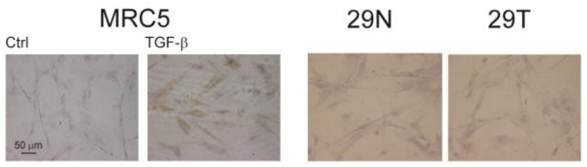

e

Sample	Cell number	Medium	Tumorigenicity
21 Tum	5000	Matrigel	3/3
	2000	IMDM	11/11
26 Tum	5000	Matrigel	2/2
	1000	IMDM	3/3
32 Tum	3000	IMDM	13/14
	1000	Matrigel	2/3
21 T-MSC	200000	Matrigel	0/3
26 T-MSC	100000	Matrigel	0/3
32 T-MSC	300000	Matrigel	0/3

f

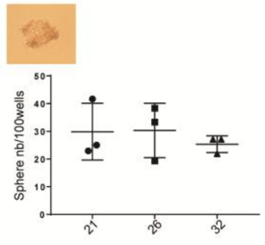

Figure S1. Characterization of primary MSC and tumor samples. Related to Figure 1

(A) Dot plots and histograms from patients 12, 16, 19, 26, 27 and 28 depicting the sequential gating strategy on living, Lin^- and $CD90^+CD105^+$ cells for the assessment of MSC-like cell proportions in normal (left panels) and tumor tissues (right panels). Percentages of Lin^-, CD90 and CD105 double positive cells, and $CD73^+$ cells among the parental population are reported. CD73 expression by $CD90^+CD105^+$ cells (blue) was calculated using unstained living cells as negative control (red). (B) Left panel: comparison of MSC proportions among Lin^- cells in normal and tumor lung samples from patients diagnosed either with Small Cell Lung Carcinoma (SCLC; n=2), Squamous Cell Carcinoma (SCC; n=3) or Adenocarcinoma (AC; n=2). Means are depicted by horizontal lines. Right panel: proportions of Lin^+ cells ($CD45^+CD34^+CD20^+CD14^+$) in 7 paired normal and tumor lung samples (connected with a line) were considered as representing the immune and endothelial cell infiltrates. (C) Clinical data (gender and age) of healthy donors from which we isolated BM-MSCs. (D) Alpha-SMA immunostaining of N- and T- MSCs from patient 29. MRC5 cells treated by TGF-β1 were used as positive control. Scale bar = 50 μm. (E) Results from mouse injections with tumor cells (Tum) or T-MSCs alone from patients 21, 26 and 32. Numbers of injected cells per mouse and medium of injection are specified. Tumorigenicity is reported as the ratio between the number of mice with tumor on the total number of injected mice. (F) Clonogenic assay of primary tumor cells from patients 21, 26 and 32 and a picture of one representative sphere counted in 32 tumor cell assay (40x magnification). For each tumor, experiments were done in triplicate. Means and standard deviations (SD) are reported.

Figure S2

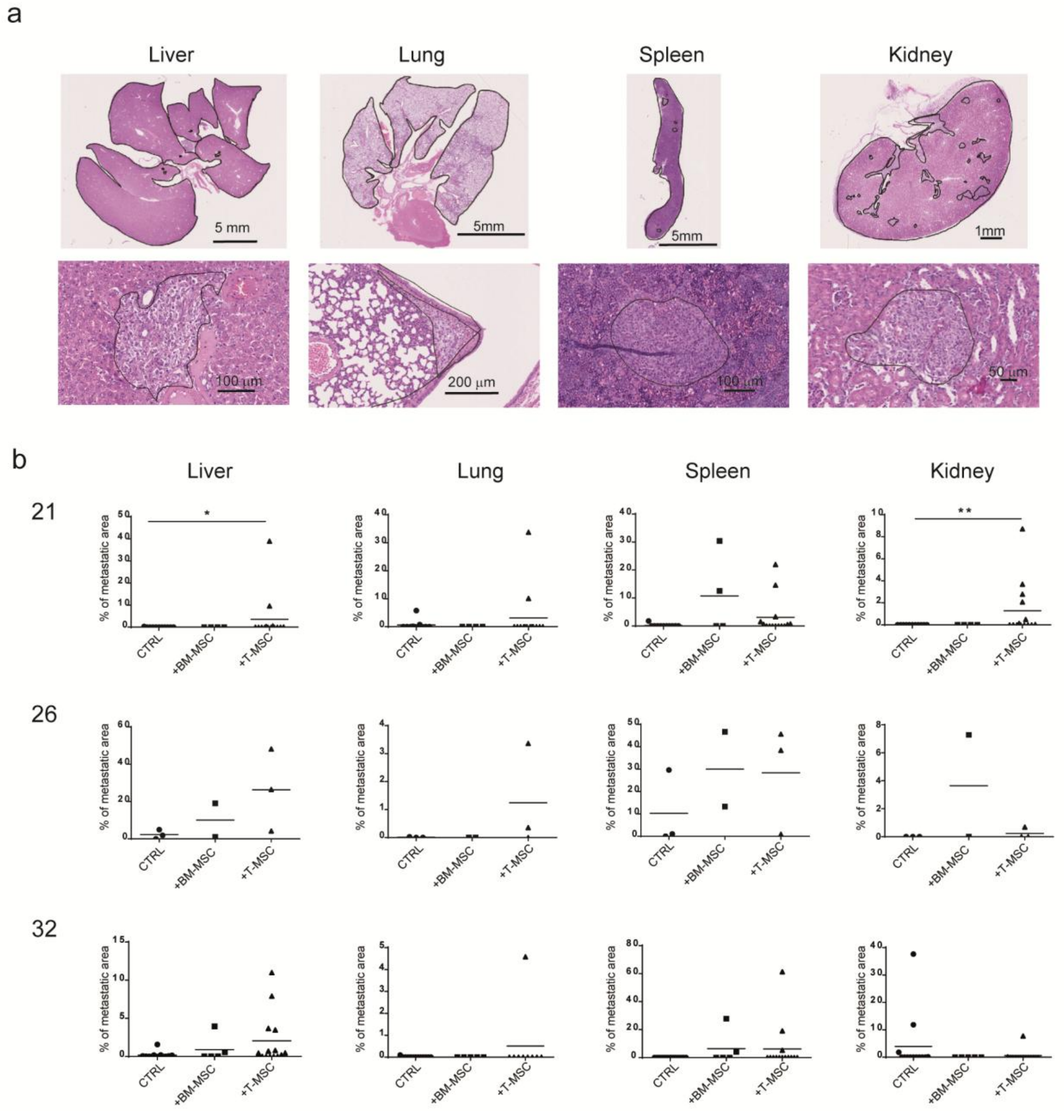

Figure S2. Metastasis quantification in liver, lung, spleen and kidney. Related to Figure 2

(A) Pictures of representative organs and metastases (from one 21 tumor +T-MSC injected mouse) as used for the metastasis quantification with the NDP.view 2 software after image acquisition by the NDP slide scanner. For quantification, circles were drawn around organs and metastases and areas automatically calculated by the software. Scale bars are reported for each picture. (B) Results represent the proportions of metastatic area per mouse for each organ in the three groups of injected mice with 21, 26 and 32 tumor cells: tumor cells alone (CTRL; n=11, 3, 13 mice, respectively), or in presence of donor 1 BM-MSCs (n=4, 2, 5 mice, respectively), or paired T-MSCs (n=14, 3, 14 mice respectively). For 21 and 32 tumor cell co-injection with MSCs, 2 experiments were performed independently and data pooled together. Means are depicted by horizontal lines. Groups were compared using the nonparametric Kruskal–Wallis (K–W) test with post-hoc Dunn's multiple comparison test. Significant adjusted p-values are indicated as * at $P \leq 0.5$, or ** at $P \leq 0.01$.

Figure S3

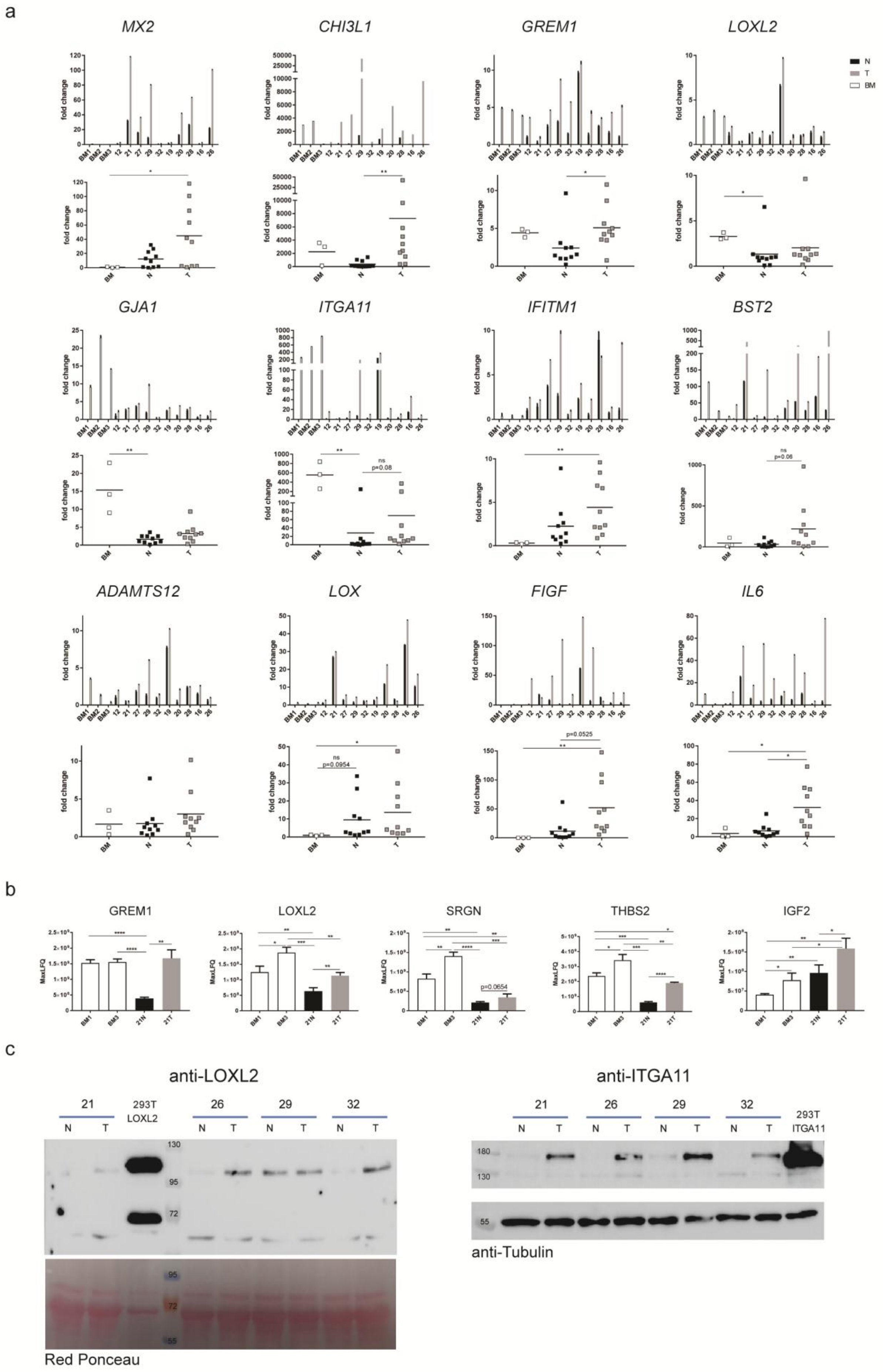
a
MX2
CHI3L1
GREM1
LOXL2
N
T
BM
fold change
GJA1
ITGA11
IFITM1
BST2
ns
p=0.08
p=0.06
ADAMTS12
LOX
FIGF
IL6
p=0.0954
p=0.0525
b
GREM1
LOXL2
SRGN
THBS2
IGF2
MaxLFQ
BM1
BM3
21N
21T
p=0.0654
c
anti-LOXL2
21
293T
LOXL2
26
29
32
N
T
130
95
72
55
Red Ponceau
anti-ITGA11
293T
ITGA11
180
130
55
anti-Tubulin

Figure S3. Comparison of gene expression levels and secretomes between BM-, N- and T-MSCs and western blot validation of differentially expressed genes. Related to Figure 3

(A) Comparison of gene expression levels of selected genes between BM- (white symbols), N- (dark symbols) and T-MSCs (grey symbols). Levels of expression were normalized on N-MSCs from patient 12 using the *PPIA* housekeeping gene (fold gene=1). Comparison of gene expressions between the three groups of samples was performed using the nonparametric Kruskal-Wallis (K-W) test with post-hoc Dunn's multiple comparison test. Significant adjusted p-values are indicated as * at $P \leq 0.5$, ** at $P \leq 0.01$. Non significant differences ($P<0.1$) are indicated as “ns” and p-values are shown. (B) Secreted levels of GREM1, LOXL2, SRGN, THBS2 and IGF2 were assessed by secretome analysis in concentrated supernatants (SN) from BM1, BM3 and compared to SN from N- and T-MSCs from patient 21. Levels of MaxLFQ (accurate proteome-wide label-free quantification by delayed normalization and maximal peptide extraction) are shown. Mean values and SD from triplicate assays are shown. The quantity of each secreted protein was compared among samples using the multiple t-test. Significant differences are indicated as reported above. (C) LOXL2 and ITGA11 expressions were assessed by western blot respectively in the SNs and cell lysates of N- and T-MSCs from patients 21, 26, 29 and 32. Membrane stained with Red Ponceau was used as equal loading control of SNs, while anti-Tubulin staining was performed as control for cell lysates. Molecular weights are reported. As positive controls for LOXL2 and ITGA11 detection, SN and cell lysate of 293T cells overexpressing each protein were used.

Figure S4

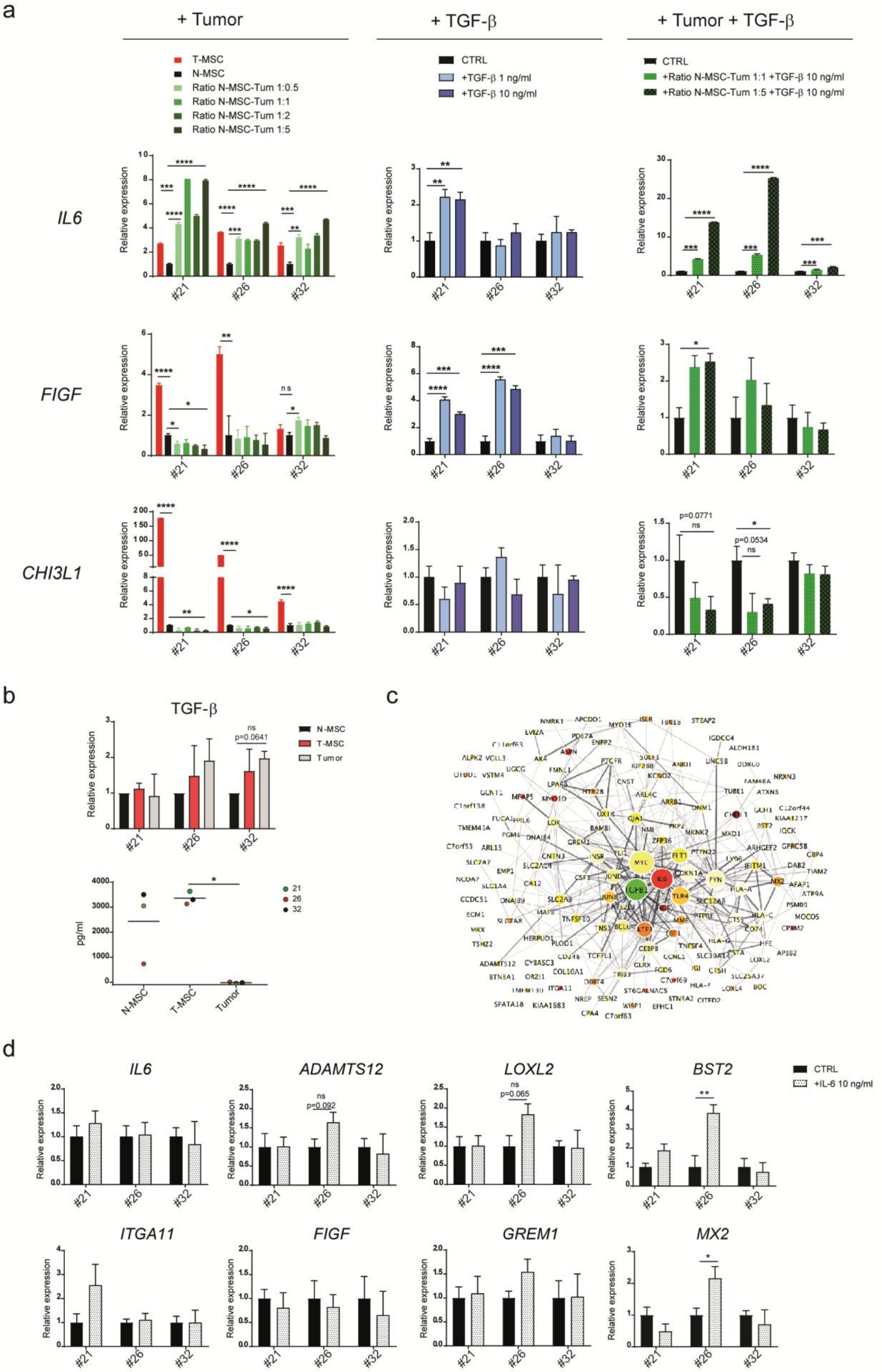

a
+ Tumor
+ TGF-β
+ Tumor + TGF-β
T-MSC
N-MSC
Ratio N-MSC-Tum 1:0.5
Ratio N-MSC-Tum 1:1
Ratio N-MSC-Tum 1:2
Ratio N-MSC-Tum 1:5
CTRL
+TGF-β 1 ng/ml
+TGF-β 10 ng/ml
+Ratio N-MSC-Tum 1:1 +TGF-β 10 ng/ml
+Ratio N-MSC-Tum 1:5 +TGF-β 10 ng/ml
IL6
FIGF
CHI3L1
Relative expression
#21
#26
#32
p=0.0771
p=0.0534
ns
b
TGF-β
N-MSC
T-MSC
Tumor
p=0.0641
pg/ml
21
26
32
c
d
IL6
ADAMTS12
LOXL2
BST2
ITGA11
FIGF
GREM1
MX2
CTRL
+IL-6 10 ng/ml
p=0.092
p=0.065

Figure S4. N-MSC phenotype is modulated by tumor microenvironment. Related to Figure 4

(A) (Left column) N-MSCs from patients 21, 26 and 32 were cultured for 7 days alone (black bars) or with increasing numbers (green color scale bars) of primary tumor cells from the same patient; N-MSC:Tumor cell ratios are reported in the legend. The expression of the reported genes was assessed by qRT-PCR and normalized to N-MSCs cultured alone using the *PP1A* housekeeping gene (fold change=1). T-MSCs alone cultured in the same conditions (red bars) are included as a reference. Median values and SD from triplicate assays are shown. For statistical analysis, N-MSCs alone were compared with T-MSCs, or with N-MSCs in the presence of the lowest or the highest amount of tumor cells. (Middle column) N-MSCs were treated for 7 days by TGF-β1 at 1 ng/ml (light blue bars) or 10 ng/ml (dark blue bars). The indicated gene expression levels are normalized to untreated N-MSCs (black bars) using the *TBP* housekeeping gene (fold change=1). Median values and SD from triplicate assays are shown. (Right column) N-MSCs from patients 21, 26 and 32 were cultured for 7 days alone (black bars) or with primary tumor cells from the same patient at 1:1 (light green bars with black dots) or 1:5 (dark green bars with black dots) N-MSC:tumor cell ratios and treated by TGF-β1 at 10 ng/ml. The expression of the reported genes was assessed by qRT-PCR and normalized to untreated N-MSCs cultured alone using the *TBP* housekeeping gene (fold change=1). Median values and SD from triplicate assays are shown.

For all experiments, gene expression levels were compared using multiple t tests using the Holm-Sidak correction method. The adjusted p-values from the comparisons of N-MSCs alone and/or untreated with N-MSCs cultured in other conditions are indicated according to level of significance: * at $P \leq 0.05$; ** at $P \leq 0.01$; *** at $P \leq 0.001$; **** at $P \leq 0.0001$. When almost significant ($P \leq 0.1$), p-values are also reported with the symbol “ns” (not significant).

(B) Gene expression levels (upper graph) and supernatant concentrations (pg/ml, lower graph) of TGF-β1 by N-, T-MSCs and tumor cells from patients 21, 26 and 32 cultured in MSC medium. For each patient, gene expression levels were normalized to N-MSCs (fold change=1) and compared by multiple t-test. Mean values and SD from two independent experiments are shown. Secretion levels of TGF-β were assessed in duplicate and mean values are shown. Comparison of TGF-β concentration in supernatants from N-, T-MSCs and tumor cells were compared by Kruskal-Wallis (K-W) test with post-hoc Dunn's multiple comparison test. Differences between groups are indicated as reported above. (C) Interaction network between TGF-β1 and proteins encoded by genes upregulated by T-MSCs identified on microarray as analyzed using Cytoscape software. Node sizes correspond to connection levels between genes, colors represent fold-change in expression (more red, higher change). Connection sizes correspond to interaction reliability in STRING. (D) N-MSCs were treated for 7 days by IL-6 (10 ng/ml, black dot filled white bars). The level of expression of the reported genes are shown and normalized to untreated N-MSCs (black bars) using the *TBP* housekeeping gene. Median values and SD from triplicates are reported. For each tumor, gene expression levels between untreated and treated N-MSCs were compared using multiple t tests and adjusted p-values indicated as described in (A).

Figure S5

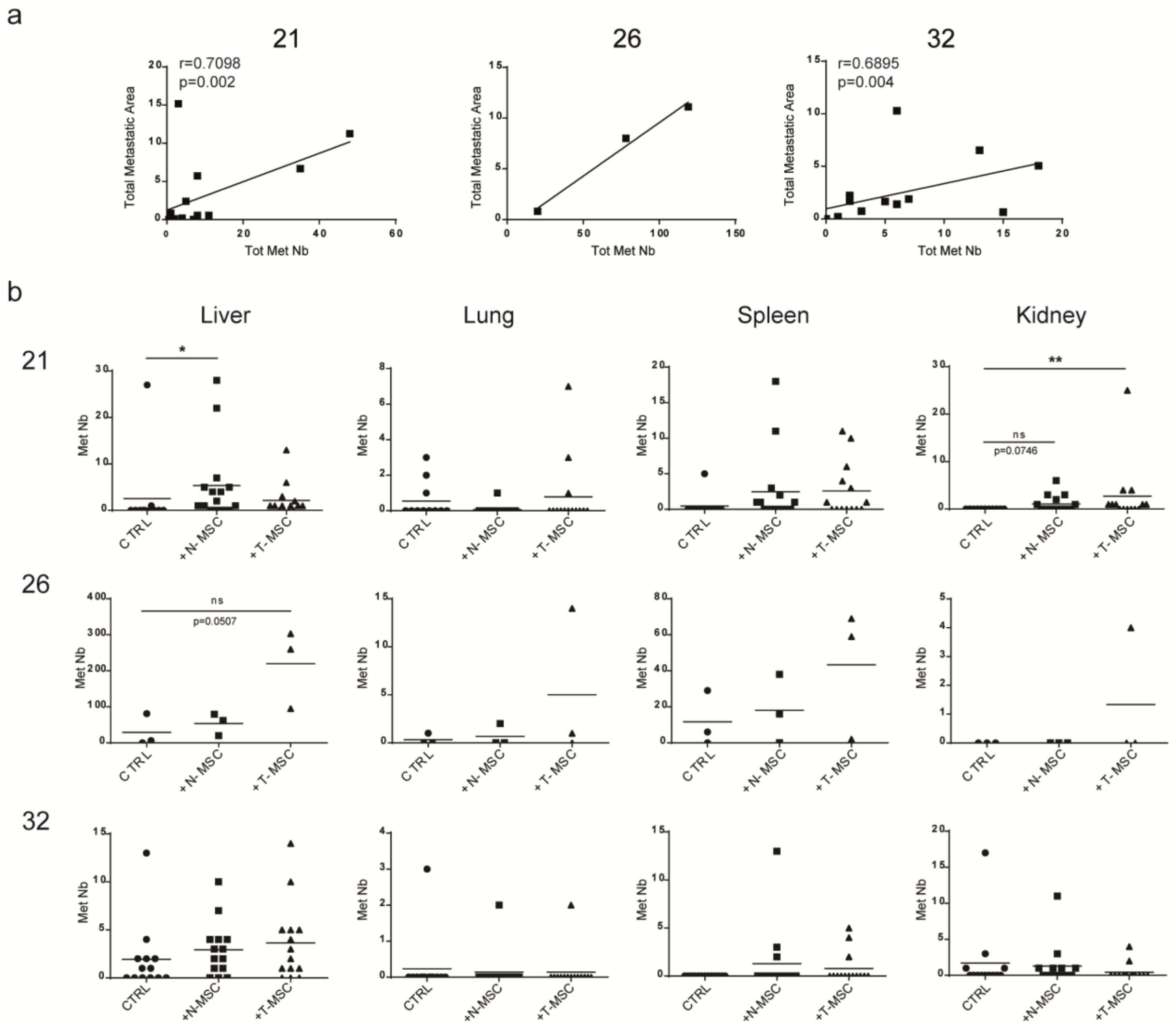

Figure S5. Metastasis quantification from mice injected with paired N- and T-MSC cells. Related to Figure 5A
(A) Correlation between total number of metastases and total metastatic area from mice injected with 21, 26 or 32 tumor cells and paired N-MSCs. Regression lines are depicted for all graphs. When possible, Spearman correlation analysis was assessed and r and p-values reported. (B) Results represent the number of metastases per mouse for each organ (liver, lung, spleen or right kidney), in the three groups of injected mice with 21, 26 and 32 tumor cells: tumor cells alone (CTRL; n=11, 3 and 13 mice respectively) or co-injected with paired N- (n=15, 3, 14 mice respectively) or T-MSCs (n=14, 3, 14 mice respectively). For 21 and 32 tumor cell co-injection with MSCs, 2 independent experiments were performed and data pooled together. Means are depicted by horizontal lines. Groups were compared using the nonparametric Kruskal-Wallis (K-W) test with post-hoc Dunn's multiple comparison test. When significant, adjusted p-values are indicated as * at P≤0.5, or ** at P≤0.01. When almost significant, adjusted p-values are also reported with the symbol “ns” (not significant.

Figure S6

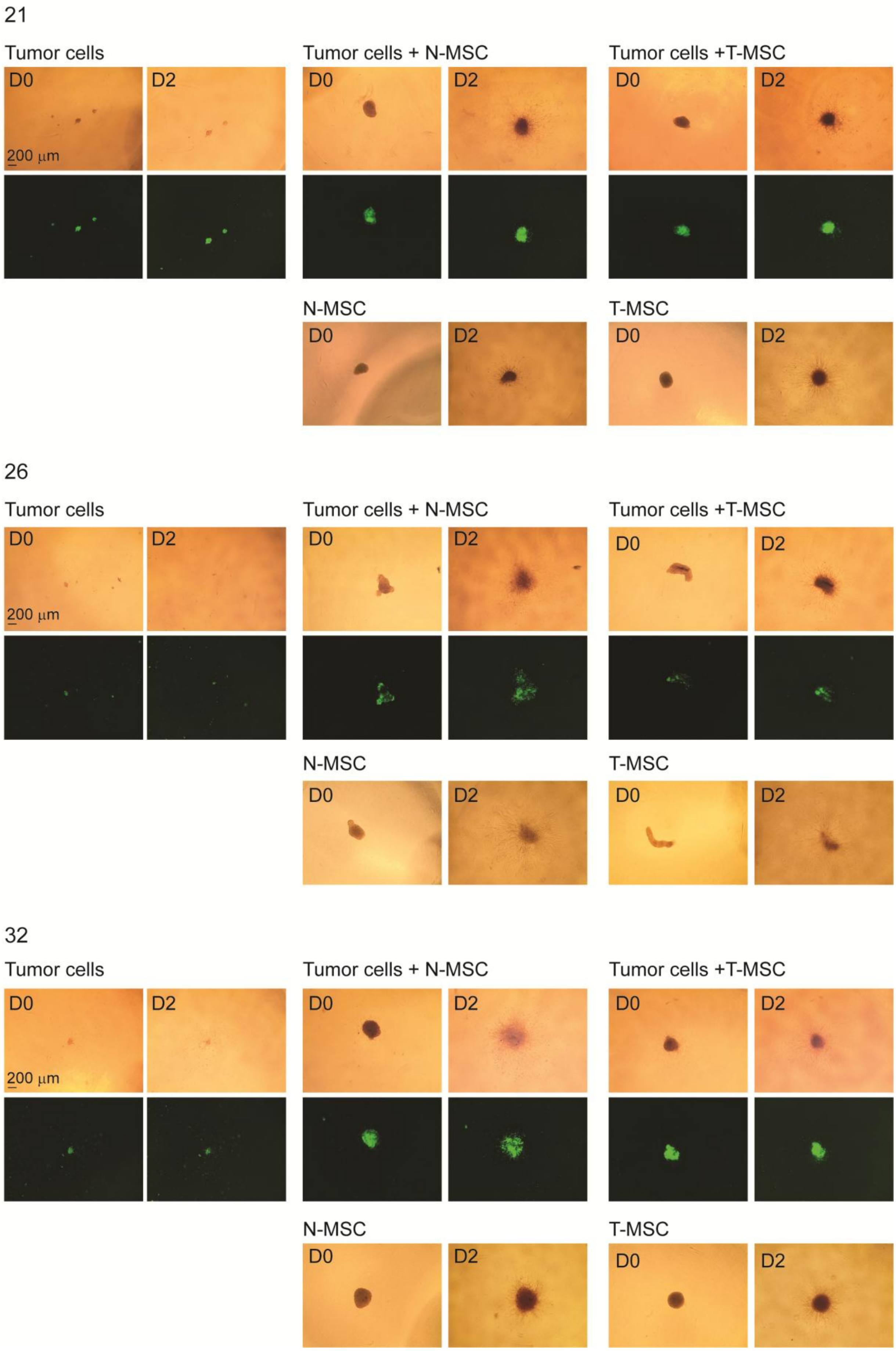
21
Tumor cells
Tumor cells + N-MSC
Tumor cells +T-MSC
D0
D2
200 μm
N-MSC
T-MSC
26
32

Figure S6. MSC:tumor cell interactions in three dimensional structures. Related to figure 5B
CFSE-labeled tumor cells from patients 21, 26 and 32 were cultured for 60 hours in hanging-drop plates alone or mixed with N- or T-MSCs at 1:1 tumor:MSC cell ratio to allow spheroid formation. N- and T-MSCs alone were cultured in the same conditions as a supplemental control. Spheroids were then included in invasion matrix. Cell dissemination and three dimensional spheroid structure was followed by microscopy and the experiment performed in quadruplicate. One representative spheroid for each condition is shown at day 0 and day 2 (after inclusion in invasion matrix) and images taken at 4x magnification by light and fluorescent microscopy. Scale bar = 200 μm is reported.

Figure S7

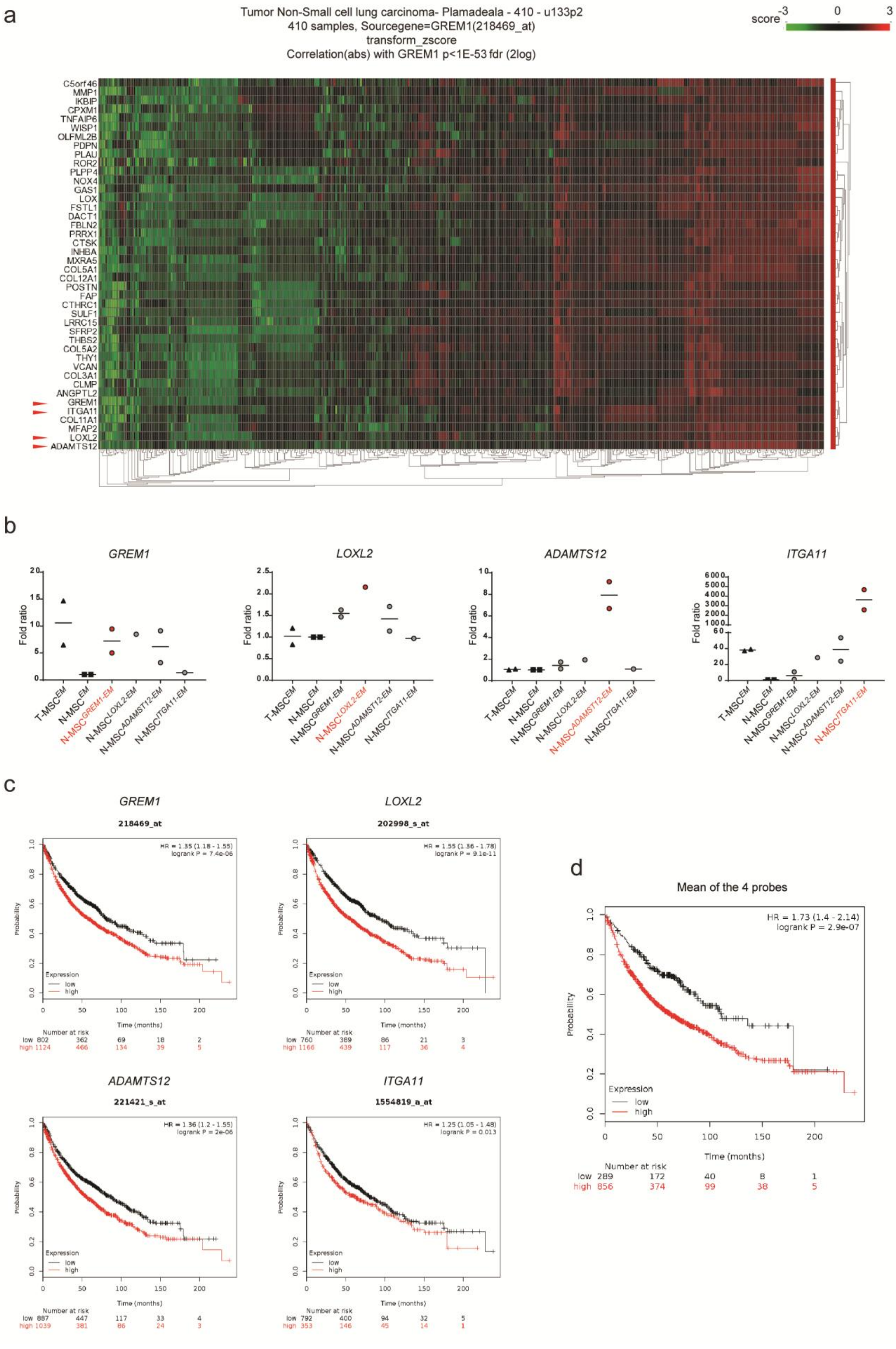

a
Tumor Non-Small cell lung carcinoma- Plamadeala - 410 - u133p2
410 samples, Sourcegene=GREM1(218469_at)
transform_zscore
Correlation(abs) with GREM1 p<1E-53 fdr (2log)
score -3 0 3
C5orf46
MMP1
IKBIP
CPXM1
TNFAIP6
WISP1
OLFML2B
PDPN
PLAU
ROR2
PLPP4
NOX4
GAS1
LOX
FSTL1
DACT1
FBLN2
PRRX1
CTSK
INHBA
MXRA5
COL5A1
COL12A1
POSTN
FAP
CTHRC1
SULF1
LRRC15
SFRP2
THBS2
COL5A2
THY1
VCAN
COL3A1
CLMP
ANGPTL2
GREM1
ITGA11
COL11A1
MFAP2
LOXL2
ADAMTS12
b
GREM1
LOXL2
ADAMTS12
ITGA11
Fold ratio
T-MSC^EM
N-MSC^EM
N-MSC^GREM1-EM
N-MSC^LOXL2-EM
N-MSC^ADAMST12-EM
N-MSC^ITGA11-EM
c
GREM1
218469_at
HR = 1.35 (1.18 - 1.55)
logrank P = 7.4e-06
LOXL2
202998_s_at
HR = 1.55 (1.36 - 1.78)
logrank P = 9.1e-11
ADAMTS12
221421_s_at
HR = 1.36 (1.2 - 1.55)
logrank P = 2e-06
ITGA11
1554819_a_at
HR = 1.25 (1.05 - 1.48)
logrank P = 0.013
Probability
Time (months)
Expression
low
high
Number at risk
d
Mean of the 4 probes
HR = 1.73 (1.4 - 2.14)
logrank P = 2.9e-07
Probability
Time (months)
Expression
low
high
Number at risk
low 289 172 40 8 1
high 856 374 99 38 5

Figure S7. *GREM1*, *LOXL2*, *ADAMTS12* and *ITGA11* mutual expression correlation and patient survival in lung carcinoma patients. Related to Figure 6

(A) Microarray heatmap of genes correlated with *GREM1* (218469_at probe) with a R p-value cutoff of 1E-53 from the dataset "Tumor Non-small cell lung carcinoma - Plamadeala - 410 - MAS5.0 - u133p2" (GEO ID: GSE63074) assessed on R2 genomics site (https://hgserver1.amc.nl/cgi-bin/r2/main.cgi). Fold changes in expression are indicated by a color scale: up-regulated genes are depicted in red, down-regulated genes in green. Each column represents a lung tumor sample while rows represent genes identified by symbols on the left of the heatmap. *GREM1*, *ITGA11*, *LOXL2* and *ADAMTS12* genes are identified by red triangles. (B) *GREM1*, *LOXL2*, *ADAMTS12* and *ITGA11* gene expression by 26T- or 26N-MSCs overexpressing the *Emerald* reporter gene (MSC^{EM}) or 26N-MSCs overexpressing also *GREM1*, *LOXL2*, *ADAMTS12*, or *ITGA11* gene. The sample infected to overexpress the gene assessed by real-time is depicted in red. Data were normalized on N-MSC^{EM} expression levels using *GAPDH* housekeeping gene. Data from two independent experiments are represented by median values of expression and means from the two experiments depicted by horizontal lines. (C) Kaplan-Meier survival plots (overall survival) for the selected genes (names of selected probes are reported) obtained from transcriptome data from patients with non-small cell lung cancer from the www.kmplot.com/lung site (version 2015; (Győrffy et al., 2013)). (D) A graph with the mean expression of the 4 selected probes was also obtained. (C-D) For each graph, hazard ratio (HR) and log-rank p-value are reported. The number of patients included at the starting point and still alive after 50, 100, 150 and 200 months from diagnosis are specified below each graph. Patients were split by medians auto-selecting the best cutoff.

Table S1. Top-50 most significantly up-regulated genes by T-MSCs. Related to Figure 3A

	**Gene Description**	**p-value**	**q-value**	**Fold change**
MX2	myxovirus (influenza virus) resistance 2 (mouse)	0.00003	0.112	1.89
FUCA1	fucosidase, alpha-L- 1, tissue	0.00015	0.113	1.29
APBB2	amyloid beta (A4) precursor protein-binding, family B, member 2	0.00015	0.113	1.18
CHI3L1	chitinase 3-like 1 (cartilage glycoprotein-39)	0.00016	0.113	9.36
NFE2L3	nuclear factor (erythroid-derived 2)-like 3	0.00018	0.113	1.35
GREM1	gremlin 1	0.00021	0.113	1.28
C11orf63	chromosome 11 open reading frame 63	0.00026	0.113	1.49
FGD6	FYVE, RhoGEF and PH domain containing 6	0.00026	0.113	1.51
LOXL2	lysyl oxidase-like 2	0.00028	0.113	1.22
MME	membrane metallo-endopeptidase	0.00030	0.113	1.87
GJA1	gap junction protein, alpha 1, 43kDa	0.00034	0.113	1.56
MYO1D	myosin ID	0.00048	0.125	2.40
GCH1	GTP cyclohydrolase 1	0.00050	0.125	1.32
PPIL6	peptidylprolyl isomerase (cyclophilin)-like 6	0.00051	0.125	1.31
KCND2	potassium voltage-gated channel, Shal-related subfamily, member 2	0.00059	0.137	1.85
ITGA11	integrin, alpha 11	0.00065	0.142	3.29
IFITM1	interferon induced transmembrane protein 1	0.00073	0.146	1.66
BOC	Boc homolog (mouse)	0.00083	0.146	1.59
CYBASC3	cytochrome b, ascorbate dependent 3	0.00086	0.146	1.38
TIAM2	T-cell lymphoma invasion and metastasis 2	0.00096	0.146	1.41
ALPK2	alpha-kinase 2	0.00101	0.146	1.65
SLC7A8	solute carrier family 7 (amino acid transporter light chain, L system), member 8	0.00115	0.146	1.97
MOCOS	molybdenum cofactor sulfurase	0.00129	0.146	1.42
TNFSF10	tumor necrosis factor (ligand) superfamily, member 10	0.00129	0.146	1.66
C7orf63	chromosome 7 open reading frame 63	0.00130	0.146	1.51
HTR2B	5-hydroxytryptamine (serotonin) receptor 2B, G protein-coupled	0.00132	0.146	1.87
BST2	bone marrow stromal cell antigen 2	0.00133	0.146	1.73
ADAMTS12	ADAM metallopeptidase with thrombospondin type 1 motif, 12	0.00133	0.146	1.69
INSR	insulin receptor	0.00141	0.146	1.29
BTN3A2	butyrophilin, subfamily 3, member A2	0.00146	0.146	1.38
MYC	v-myc myelocytomatosis viral oncogene homolog (avian)	0.00146	0.146	1.45
KIAA1217	KIAA1217	0.00152	0.146	1.66
TBX18	T-box 18	0.00153	0.146	1.98
IQCK	IQ motif containing K	0.00159	0.146	1.30
MXD1	MAX dimerization protein 1	0.00163	0.146	1.23
LOX	lysyl oxidase	0.00166	0.146	1.32
CPXM2	carboxypeptidase X (M14 family), member 2	0.00167	0.146	3.14
FIGF	c-fos induced growth factor (vascular endothelial growth factor D)	0.00168	0.146	5.11
NMI	N-myc (and STAT) interactor	0.00168	0.146	1.18
NREP	neuronal regeneration related protein homolog (rat)	0.00177	0.146	1.24
ARL4C	ADP-ribosylation factor-like 4C	0.00182	0.146	1.30
CCDC51	coiled-coil domain containing 51	0.00183	0.146	1.17
TRIB3	tribbles homolog 3 (Drosophila)	0.00197	0.150	1.25
NRXN3	neurexin 3	0.00198	0.150	1.89
ENPP2	ectonucleotide pyrophosphatase/phosphodiesterase 2	0.00203	0.150	1.54
EBF1	early B-cell factor 1	0.00221	0.157	1.99
SLC12A8	solute carrier family 12 (potassium/chloride transporters), member 8	0.00230	0.157	1.53
CDKN1A	cyclin-dependent kinase inhibitor 1A (p21, Cip1)	0.00231	0.157	1.22
OXTR	oxytocin receptor	0.00235	0.157	1.64
IL6	interleukin 6 (interferon, beta 2)	0.00252	0.161	2.81

Table S2. Up- and down- regulated genes by tumor cells following MSC co-culture. Related to Figure 6A-B

Tumor sample	Culture condition[a]	Gene list[b]	Modulation
21	+N-MSCs	CCDC73, IL12B, KALRN, KRT5, GHR, IL1R2, MAL, PRRX1, SSTR2, USP51, WNT7B	DOWN
	+T-MSCs	CCDC73, IL12B, KALRN, KRT5, CCR4, CXCL9, DFNB59, DGKI, FGF5, KCNMB1, SERPINB10, SRGAP1	
	+N-MSCs	ADM, AK1, APLP1, ARG2, ARVCF, ATP4A, BHLHE41, C10orf99, CCND1, CD101, CD180, CD96, CEACAM1, COL6A2, COL7A1, CPLX1, DHRS9, EFEMP2, FAM13A, FFAR2, FKBP9, GAS6, GDF15, GLIS2, LGALS9C, LOC101927412, LPIN3, LRP1, MAFF, MAP1A, MMP14, MXI1, MYBPC2, PGF, PLD4, PLEKHB1, PLK2, PTPRO, RAB37, RARRES3, RRAD, SERPINE1, SMPDL3B, TBX15, TEX19, TGM2, TNFRSF10D, TPM2, TSPAN9, UNC13A, VEGFA, ZNF385A, BATF2, CD1C, ENAM, FAM129B, LRRC25, LRRC31, MAP7D2, MFSD7, MMP1, MMP2, NES, PHLDA2, PTRF, S100A6, SERPINA1	UP
	+T-MSCs	ADM, AK1, APLP1, ARG2, ARVCF, ATP4A, BHLHE41, C10orf99, CCND1, CD101, CD180, CD96, CEACAM1, COL6A2, COL7A1, CPLX1, DHRS9, EFEMP2, FAM13A, FFAR2, FKBP9, GAS6, GDF15, GLIS2, LGALS9C, LOC101927412, LPIN3, LRP1, MAFF, MAP1A, MMP14, MXI1, MYBPC2, PGF, PLD4, PLEKHB1, PLK2, PTPRO, RAB37, RARRES3, RRAD, SERPINE1, SMPDL3B, TBX15, TEX19, TGM2, TNFRSF10D, TPM2, TSPAN9, UNC13A, VEGFA, ZNF385A, ADAP2, AMBP, ANKRD65, APOBEC3H, ARHGEF40, ATF3, BBC3, C10orf10, C2, C6orf52, CA11, CAPN12, CD68, CDC14B, CDC42BPG, CEL, CERCAM, COPZ2, CROCC, CRYM, CTSF, CYP4F2, DDIT4, DHRS3, DPEP2, DQX1, DUSP8, EPHA2, EPHX1, EPS8L2, ESPNL, FFAR1, FGF11, FLJ20021, FSD1, GDPD5, GLS2, HSD17B14, KCNJ15, LCE1E, LINC00908, LINC01358, LINC01530, LRG1, MFGE8, MIR210, MYO15B, NDRG1, NDUFA4L2, NKG7, NUAK2, PCAT6, PDE6G, PDZD2, PLEKHN1, PLXNB3, PODXL, PPFIA4, PPM1J, PPP1R32, PVRL4, PXT1, RAB11FIP5, RAB26, RAP1GAP2, SAT1, SDC3, SLC6A8, SPAG4, SPON2, SSPN, SSTR3, STRC, THRA, TNN, TNNT3, TXNIP, WNT10B, WNT11, ZCCHC24	
32	+N-MSCs	CXCL14, ENTPD2, FRMPD2, KALRN, C17orf99, CDH24, COL5A1, DNASE1L3, EEF1A2, FUT7, IL1B, KCNMA1, KIF26B, LOC100130476, MT1H	DOWN
	+T-MSCs	CXCL14, ENTPD2, FRMPD2, KALRN, JPH4	
	+N-MSCs	C3, NAPSA, AK4, ANKRD34A, BMP7, BNIP3, BSPRY, CABP1, CCNG2, CD180, CORO2A, CUEDC1, DARS-AS1, DHRS3, DOK2, EML1, FGF11, FGFR3, FUT11, GDF15, GLUL, GPR146, HILPDA, HK2, MACROD2, MAP7D2, NRN1, P4HA1, PCYT1B, PDE4C, PEX11A, PLEKHB1, RNASET2, SDC3, SEMA6A, SHC4, SLC2A3, SLC2A5, SLC6A20, SPAG4, SPRY1, TGFBR3, TGM2, TMEM45A, VEGFA, ZNF385A	UP
	+T-MSCs	C3, NAPSA	

[a] Results from tumor co-cultures with paired or 29 MSCs were pooled together and generally indicated as "+N-MSCs" or "T-MSCs"; [b] in blue, overlapping up- or down- regulated genes after tumor co-culture with N- and T-MSCs

Table S3. *ADAMTS12*, *LOXL2*, *GREM1*, *ITGA11* gene expression correlation in microarray datasets from CAFs[a] and MSCs[b] isolated from primary lung carcinoma samples. Related to Figure 7 and S7

CAF in NSCLC[c] (GSE22874)

Correlation	*ADAMTS12*	*LOXL2*	*GREM1*	*ITGA11*
ADAMTS12	1.000	0.499	0.408	0.691
LOXL2	0.499	1.000	0.482	-0.102
GREM1	0.408	0.482	1.000	0.093
ITGA11	0.691	-0.102	0.093	1.000

P-value	*ADAMTS12*	*LOXL2*	*GREM1*	*ITGA11*
ADAMTS12	0.000	0.058	0.130	0.004
LOXL2	0.058	0.000	0.069	0.720
GREM1	0.130	0.069	0.000	0.740
ITGA11	0.004	0.720	0.740	0.000

NSCLC-MSC (GSE23066)

Correlation	*ADAMTS12*	*LOXL2*	*GREM1*	*ITGA11*
ADAMTS12	1.000	0.965	0.955	0.675
LOXL2	0.965	1.000	0.997	0.784
GREM1	0.955	0.997	1.000	0.824
ITGA11	0.675	0.784	0.824	1.000

P-value	*ADAMTS12*	*LOXL2*	*GREM1*	*ITGA11*
ADAMTS12	0.000	0.007	0.011	0.211
LOXL2	0.007	0.000	0.000	0.117
GREM1	0.011	0.000	0.000	0.086
ITGA11	0.211	0.117	0.086	0.000

T-MSC[d] **in lung carcinoma** (our dataset; GSE104636)

Correlation	*ADAMTS12*	*LOXL2*	*GREM1*	*ITGA11*
ADAMTS12	1.000	0.680	0.861	0.820
LOXL2	0.680	1.000	0.531	0.510
GREM1	0.861	0.531	1.000	0.815
ITGA11	0.820	0.510	0.815	1.000

P-value	*ADAMTS12*	*LOXL2*	*GREM1*	*ITGA11*
ADAMTS12	0.000	0.044	0.003	0.007
LOXL2	0.044	0.000	0.141	0.161
GREM1	0.003	0.141	0.000	0.007
ITGA11	0.007	0.161	0.007	0.000

[a]CAF: carcinoma-associated fibroblasts; [b]MSC: mesenchymal stem cells; [c]NSCLC: non-small cell lung carcinoma; [d]T-MSC: tumor-associated mesenchymal stem cells; significant results ($p<0.05$) are highlighted in yellow.

Supplemental Experimental procedures

Flow cytometry analyses

For all staining, cells were first incubated 15 minutes with FcR Blocking Reagent (Miltenyi Biotech), washed once in PBS1x (Bichsel AG, Interlaken CH) and stained 30 min at 4°C with conjugated antibodies (Abs) and live/dead marker (1:200, Violet fluorescent reactive dye, Invitrogen) diluted in PBS1x and finally fixed with PBS1x/PFA1%. For some sample, dead cell exclusion was performed using DAPI staining (10 µg/ml, Biotium) on non-fixed samples. Electronic compensation was set up using CompBeads (Beckman Coulter) to correct for fluorochrome spectral overlap. All samples were acquired with Gallios Cytometer (Beckman Coulter) and data analyzed using FlowJo version10 software following doublet and dead cell exclusion.

MSC % in normal and tumor tissues. After tissue dissociation, MSC proportions were assessed on 10^6 single cells from tumor bulk and normal tissues using the MSC phenotyping kit (1:10, Miltenyi). A minimum amount of 10^5 living cells was acquired. MSC-like cells were defined as $CD90^+CD105^+CD73^+$ and their percentage calculated among Lin^- cells ($CD45^-CD34^-CD20^-CD14^-$) after gating on living cells. The proportion of Lin^+ cells among living cells was considered as a broad quantification of the immune cell infiltrate ($CD45^+CD20^+CD14^+$ cells) and lung endothelial cells ($CD34^+$ cells) (Müller et al., 2002) in tumor tissues. Cells stained with DAPI or live/dead marker in absence of Abs were used as control condition.

MSC phenotype. All MSC cell cultures were analyzed for the expression of CD90 (1:10, FITC-conjugated, Miltenyi Biotech), CD166 (1:20, PerCP-Cy5.5-conjugated, BD Pharmingen), CD105 (1:10, PE-conjugated, Miltenyi Biotech), CD44 (1:20, APC-H7-conjugated, BD Pharmingen), CD73 (1:10, APC-conjugated, Miltenyi Biotech), CD45 (1:20, Alexa700-conjugated, BD Pharmingen) surface markers on 10^5 cells per condition. The proportion of positive cells was calculated on more than 5000 living cells after doublet exclusion. Cells stained with live/dead marker in absence of Abs were used as control condition.

Immunohistochemistry

MSCs. BM3 and N- and T-MSCs (40,000 cells/well) from patients 12, 21, 26, 27, 29, 32 were grown on cover glasses for 24h in 24-well plates, washed twice with PBS1x, fixed in PFA 4% at room temperature (RT) and cover glasses loaded on Superfrost Plus slides (ThermoFisher) using the Eukitt quick-hardening mounting medium (Fluka). Cells were blocked 10 min in normal goat serum (1:10 in PBS1x), rinsed in PBS1x and stained for one hour with primary antibodies: mouse anti-Vimentin (1:200, EDTA retrieval, Dako) or rabbit anti-alpha-SMA (1:200, no retrieval, Abcam). Cells were then washed, stained 30 min with the appropriate HRP secondary antibody, rinsed and stained 5 min with DAB+ (Dako). Cell nuclei were colored with Hematoxylin, cells rinsed and finally dehydrated with multiple washing steps in EtOH (95%-100%) and xylol. Sakura Tissue-Tek Film was then used for film coverslipping. As positive control for alpha-SMA staining, we used MRC5 cells treated for 3 days with TGF-β1 (10ng/ml, Miltenyi Biotech) in DMEM (Gibco) without serum. MRC5 grown in DMEM complemented with 10% FBS and 1% PS were used as internal control.

Tumor cells. Paraffin-embedded tumor tissues from patients 21 and 32 were single stained with mouse anti-p63 (1:100, Agilent) and mouse anti-TTF1 (1:30, Invitrogen) Abs, tissue from patient 26 with mouse anti-chromogranin A (1:200, Agilent) and anti-TTF1 Abs. Tumor sphere cultures from the same patients were fixed in PFA 4%, included in 1.5% low melting point agarose (Quantum-Appligene), fixed again, paraffin-embedded and analyzed for expression of the same markers. Tumor tissue and sphere staining were automatically performed on a Ventana instrument at the Institute of Pathology (CHUV – Lausanne, Switzerland).

MSC multi-lineage differentiation potential

All primary MSC samples were assessed for their differentiation potential along osteocytic, chondrocytic and adipocytic lineages using three different conditioned media. For induction of adipocytic and osteocytic differentiation, MSCs were seeded on cover glasses at 0.1 M cells/well in 12-well plates, in 1 ml medium/well. When MSCs reached 80% confluence, cells for adipocytic differentiation were treated with IMDM, supplemented with 1% PS, 10% FBS and 1% NEAA, ITSS (Insulin Transferin Sodium Selenite; 10 µg/ml, Roche), Dexamethasone (10^{-6} M, Sigma), Indometacine (100 µM, Fluka) and IBMX (3 Isobutyl 1 Methylxanthine, 100 µM, Sigma). For osteocytic induction, MSCs were treated with DMEM (GIBCO) supplemented with PS 1%, FBS 10%, NEAA 1%, AAP (Ascorbic Acid P; 50 µg/ml, Sigma), Dexamethasone (10^{-7} M) and bGP (betaGlycerophospate; 5 mM, Sigma). MSCs cultured in MSC medium were used as controls for both induction conditions. For chondrocytic induction, 0.5 M cells were cultured as a pellet in 1 ml medium. Induction medium was composed of DMEM (high glucose), PS 1%, ITSS (10 µg/ml) and linoleic acid (1 mg/ml, Sigma), with AAP (50 µg/ml), Dexamethasone (10^{-7} M), and TGF-β1 (10 ng/ml, Miltenyi Biotec). Control medium consisted of DMEM supplemented with PS 1%, ITSS (10 µg/ml) and linoleic acid (1 mg/ml). For all differentiation tests, cells were treated for three weeks and medium changed three times per week. Following the treatments, cells were washed in PBS, fixed in PFA 4% at RT and the cover glasses loaded on Superfrost Plus slides. Cell pellets were included in paraffin using the processor Leica ASP200S and then cut in 3 µm thick slices by the microtome Microm HM 355S. Depending on the differentiation assay, cells were then stained either with oil red O (adipocytes), silver nitrate 5% (von Kossa staining; osteocytes), or Alcian blue (chondrocytes).

Clonogenic assay

Tumor spheres were mechanically dissociated and plated at single-cell density in 96-well low-adherence plates (Corning) in 100 µl/well of KO medium. 50 µl of fresh medium were added per well every week. Sphere numbers were assessed after 4 weeks by microscopy (Nikon Eclipse TS100). For each tumor cell type, experiments were performed in triplicate.

MSC expression profile analysis by microarray

RNA quantities were assessed by NanoDrop ND-1000 spectrophotometer and RNA quality by RNA 6000 NanoChips with the Agilent 2100 Bioanalyzer (Agilent Technologies, Palo Alto, USA). For each sample, 100 ng of total RNA were amplified using the WT Expression kit (Invitrogen, Carlsbad, USA; catalogue no. 4411974); the resulting sense cDNA was fragmented using UDG (uracil DNA glycosylase) and APE 1 (apurinic/apyrimidic endonuclease 1) and biotin-labeled with TdT (terminal deoxynucleotidyl transferase) using the GeneChip WT Terminal labeling kit (Affymetrix, Santa Clara, USA; catalogue no. 900671, Santa Clara, USA). Affymetrix Human Gene 1.0 ST arrays were hybridized with 2.5µg of biotinylated target, at 45°C for 17 hours washed and stained according to the protocol described in Affymetrix GeneChip Expression Analysis Manual (Fluidics protocol FS450_0007).
The arrays were scanned using the Affymetrix GeneChip Scanner 3000 7G and raw data extracted from the scanned images and analyzed with the Affymetrix Power Tools software package.

BM-MSC expression profile analysis by qRT-PCR

For BM-MSC comparison with N- and T-MSC gene expression levels, RNA extraction and c-DNA synthesis were performed as described in “RNA extraction, cDNA synthesis and qRT-PCR” experimental procedure section. Data were normalized on expression levels of N-MSCs from patient 12 (fold change=1) using the *PPIA* housekeeping gene. Gene expression levels between BM-, N- and T-MSCs were compared using the nonparametric Kruskal-Wallis (K-W) test with Dunn’s multiple comparison test. Significant differences were indicated as *, ** according to level of significance (*, $P \leq 0.05$; **, $P \leq 0.01$). Not significant p-values ($P<0.1$) were reported and indicated as “ns” (not significant).

Primer sequences for qRT-PCR

	Forward	Reverse
ADAMTS12	5'- ATTGTTGTGGTTCGGCTCATT -3'	5'- AGGTCACTCTTGGGATTGATACT -3'
BST2	5'- CACACTGTGATGGCCCTAATG -3'	5'- GTCCGCGATTCTCACGCTT -3'
CHI3L1	5'- GTGAAGGCGTCTCAAACAGG -3'	5'- GAAGCGGTCAAGGGCATCT -3'
FIGF	5'- ATGGACCAGTGAAGCGATCAT -3'	5'- GTTCCTCCAAACTAGAAGCAGC -3'
GJA1	5'- GGTGACTGGAGCGCCTTAG -3'	5'- GCGCACATGAGAGATTGGGA -3'
GREM1	5'- CGGAGCGCAAATACCTGAAG -3'	5'- GGTTGATGATGGTGCGACTGT -3'
IFITIM	5'- CCAAGGTCCACCGTGATTAAC -3'	5'- ACCAGTTCAAGAAGAGGGTGTT -3'
IL-6	5'- ACTCACCTCTTCAGAACGAATTG -3'	5'- CCATCTTTGGAAGGTTCAGGTTG -3'
ITGA11	5'- GTGGCAATAAGTGGCTGGTC -3'	5'- GTTCCCGTGGATCACTGGAC -3'
LOX	5'- CGGCGGAGGAAAACTGTCT -3'	5'- TCGGCTGGGTAAGAAATCTGA -3'
LOXL2	5'- GGGTGGAGGTGTACTATGATGG -3'	5'- CTTGCCGTAGGAGGAGCTG -3'
MX2	5'- CAGAGGCAGCGGAATCGTAA -3'	5'- TGAAGCTCTAGCTCGGTGTTC -3'
TGF-β1	5'- CAGATCCTGTCCAAGCTG -3'	5'- TCGGAGCTCTGATGTGTT -3'
GAPDH	5'- AGCCACATCGCTCAGACAC -3'	5'- GCCCAATACGACCAAATCC -3'
TBP	5'- CGGCTGTTTAACTTCGCTTC -3'	5'- CACACGCCAAGAAACAGTGA -3'

Secretome analysis

N- and T-MSCs from patients 12 and 21 were plated at $0.5x10^6$ in $150cm^2$ dishes (Nunc) in MSC medium. When reaching 80% confluence, they were cultured in FBS-free condition for 24h in 22ml of IMDM without phenol red supplemented with PS1% and PDGF 10ng/ml. Supernatants (SN) were centrifuged 3 times to remove living cells (10’ at 300xg), dead cells (10’ at 2000xg) and debris (30’ at 10000xg) using a swinging bucket rotor in Avanti J-20 XPI centrifuge (Beckman Coulter). SN were then concentrated using Amicon Ultra-15 centrifugal filter columns (3 kDa) proceeding with subsequent centrifugation steps at 4000xg in swinging rotor and up to 1.5 ml of residual SN. SNs were then diluted in ammonium bicarbonate (100mM) up to 12ml, centrifuged again at 4000xg until 1.5ml residual volume, re-diluted in ammonium bicarbonate (100mM, 12ml) and concentrated again to a final volume of 0.5ml. All centrifugation steps were done at +4°C. Experiments were performed in triplicate and concentrated SN kept at -80°C until secretome analysis.
After buffer exchange, secretome samples were lyophilized and redissolved in buffered 8M Urea. After reduction/alkylation of cysteines, proteins were digested with 0.05 ug Trypsin (Modified, Promega) for 18h at 37C.

Digests were desalted and analysed on a high resolution hybrid LTQ Orbitrap Velos mass spectrometer (Thermo Fisher Scientific, Bremen, Germany) coupled to a nano-liquid chromatography system as previously described (Mauvoisin et al., 2014). All samples were analysed twice to improve sampling.
Data were processed with MaxQuant 1.4.1.2 (Cox and Mann, 2008; Cox et al., 2011) searching the human subset of the UNIPROT database (release 2013_09) with standard parameters (carbamidomethyl Cys, variable oxidation of methionine and acetylation of protein N-termini). Mass tolerance was 4.5 ppm on precursors (after recalibration) and 0.5 Da on CID fragments. Identifications were filtered at 1% FDR at both the peptide and protein level by comparison against a decoy database. Label-free quantitation (LFQ) of samples was performed by MaxQuant as described (Cox et al., 2011).
Subsequent analysis of LFQ values was performed using the Local-Pooled-Error statistical test ((Jain et al., 2003), R package version 1.36.0) with multiple testing correction (Benjamini and Hochberg, 1995) and threshold at 0.05.
Repeated analysis of 21N- and 21T-MSC secretomes together with BM1 and BM3 samples were prepared and processed similarly, with the only difference that MS analysis was done on a Fusion Tribrid Orbitrap instrument (Thermo Fisher Scientific, Bremen, Germany). For data analysis, the version 1.6.0.13 of the MaxQuant software was used. Multiple t tests were used to compare mean secretion levels of GREM1, LOXL2, SRGN, THBS2 and IGF2 in the supernatants of 21N, 21T, BM1 and BM3 samples. Significant differences were indicated as *, **, *** or **** according to level of significance (*, $P \leq 0.05$; **, $P \leq 0.01$; ***, $P \leq 0.001$; ****, $P \leq 0.0001$).

Western Blot
ITGA11 and LOXL2 protein expression were assessed respectively in the cell lysates and supernatants of N- and T-MSC cells from patients 21, 26, 29 and 32. For sample preparation, cells were plated in 10cm^2 petri dishes (450.000 cells/dish) in MSC medium (10ml/dish) and when reaching 70% confluence, the medium was changed and cells kept 24h in FBS-free IMDM medium. Supernatants (SN) were collected, centrifuged once at 300g to remove cells and concentrated using Amicon Ultra-15 centrifugal filter columns (3 kDa) proceeding with subsequent centrifugation steps at 4000xg in swinging rotor and up to 0.5 ml of residual SN. For protein preparation, cells were harvested with Trypsin-EDTA 0.25 mg/ml and lysed in a buffer containing Tris-HCl 50 mM pH 7.4, NaCl 150 mM, Triton 1.0%, EDTA 1mM and a protease inhibitor cocktail added fresh (complete MINI protease inhibitors, Roche). The lysate was vortexed, kept 25 min on ice and centrifuged for 10 minutes at 7300 RPM. The recovered supernatant was used for protein sample preparation.
Western blotting was performed according to standard procedures. For ITGA11 detection, 30µg of total protein were loaded per lane in Tris-glycine SDS-Polyacrylamide 8% gel, and blotted on nitrocellulose membrane (Amersham). For LOXL2 detection, 40µl of concentrated SN were loaded per lane in an 8% gel. Membranes were then blocked 1h at RT in TBST-Milk 5% and incubated over night at +4°C with primary antibodies in TBST-Milk 2.5% at the indicated dilution: rabbit anti-ITGA11 (1:1000; ref. ab198826, Abcam); rabbit anti-LOXL2 (1:1500; ref. ab96233, Abcam). Mouse anti-Tubulin (1:3000, ref. CP06-100µg, Calbiochem) was used to normalize ITGA11 expression quantity. LOXL2 in SN was normalized using Ponceau S staining. Secondary antibodies were: horseradish peroxidase (HRP)–conjugated goat anti-rabbit (ref. A0545, Sigma) and goat anti-mouse (ref. 1721011, BioRad) antibodies. For Western Blot revelation, membranes were incubated with SuperSignal West Pico Chemiluminescent Substrate (ref. 34080, Thermo Scientific) or WesternBrightSirius (ref. K12043, Witec AG) HRP Subtrate and images were acquired using Fusion FX apparatus (Vilbert Lourmat). Cell lysate and SN from 293T cells overexpressing ITGA11 and LOXL2 respectively (see "N-MSC overexpression of selected genes" section) were used as positive control.

TGF-β1 expression and secretion by MSC and tumor cells
qRT-PCR. TGF-β1 gene expression levels were assessed on tumor cells, N- and T-MSCs from patients 21, 26 and 32 cultured in MSC medium in two independent experiments. For each patient samples, results were normalized on N-MSC levels of expression using the *GAPDH* housekeeping genes. Multiple t tests were used to compare mean expression levels from the two independent experiments between tumor, N- and T-MSC samples from the same patient. Significant differences were indicated as *, **, *** or **** according to level of significance (*, $P \leq 0.05$; **, $P \leq 0.01$; ***, $P \leq 0.001$; ****, $P \leq 0.0001$).
Secretion. Tumor cells, N- and T-MSCs (400,000 cells) from patients 21, 26 and 32 were cultured in 1 ml of MSC medium for 72 hours. Supernatants were collected, centrifuged 5 minutes at 1400 rpm to remove dead cells and frozen at -80°C until TGF-β1 quantification. TGF-β1 concentrations in cell supernatants was assessed on a Luminex 200 instrument using the Milliplex MAP TGFb1 Magnetic Beads Single Plex kit (Merckmillipore; Cat# TGFBMAG-64K-01) and following manufacturer's instructions. Analyses were performed in duplicate. Kruskal-Wallis (K-W) test with Dunn's multiple comparison test was used to compare TGF-β1 secretion by N-, T-MSC and tumor cells. Significant differences were indicate as described above.

Protein interaction network
We extracted the list of genes that were upregulated (q-value<0.2) in T-MSCs compared to N-MSCs from the microarray experiment. With Cytoscape software (Shannon et al., 2003), we built a protein interaction network using STRING v10 database (Szklarczyk et al., 2015) with differential genes as nodes. The width of edges represents the degree of confidence of the interaction from STRING, the size of each node, the degree of each node (number of connections) and the color, the fold-change between the conditions.

RNA-sequencing

Total RNA was quantified with a Qubit fluorimeter (Life Technologies) and RNA integrity assessed with a Bioanalyzer (Agilent Technologies). The TruSeq mRNA stranded kit from Illumina was used for the library preparation with 150 ng (1st experiment) or 200 ng (2nd experiement) of total RNA as input. Library molarity and quality was assessed with the Qubit and Tapestation using a DNA High sensitivity chip (Agilent Technologies). Pools of 6 libraries were loaded at 8.5 pM for clustering on a Single-read Illumina Flow cell. Reads of 100 bases were generated using the TruSeq SBS HS v3 chemistry on an Illumina HiSeq 2500 sequencer. The quality was checked using FastQC v.0.11.2 and the reads were mapped with TopHat v2.0.13 (default parameters) on hg19 reference genome. Biological quality control and summarization were done with RSeQC v2.6.1 and PicardTools v1.80. The number of reads by gene was obtained using HTSeq v.0.6.1 with the command *htseq-count –mode=union, --stranded=reverse*.

Transwell invasion assay

Day 0. Tumor cells from patients 21 and 32 were starved overnight in IMDM medium supplemented with BSA 0.2% (Bovine serum albumin; New England Biolabs). Invasion chambers were prepared by coating cell culture inserts (24 well 8.0 µm pore size insert of PET-membrane; Corning, Falcon) with 60 µl of IMDM-diluted GFR matrigel 4 mg/ml (Becton Dickinson AG) and keeping the inserts overnight at 37°C to allow matrigel polymerization. Paired N- or T-MSCs (15000 cells/well) were counted, transferred in 24 well plates (Corning) in MSC medium and left overnight at 37°C to allow cell adhesion in the lower chamber. *Day 1.* Invasion chambers were prepared transferring matrigel-coated inserts to 24 well plates. According to the assay condition, lower chambers were prepared by adding 600 µl/well of IMDM medium without serum or IMDM supplemented with 10% FBS before transferring the inserts. In MSC-coated wells, medium was substituted with IMDM-FBS10% medium (600 µl/well). Starved tumor cells were stained with CFSE 10 µM following manufacturer's instructions, counted and transferred on the top (15000 cells/well) of each matrigel-coated inserts in IMDM medium without serum (200 µl/well). Invasion of tumor cells induced by the presence of MSCs and 10% FBS in the lower chambers was compared with invasion observed in the presence of IMDM medium supplemented or not with 10% FBS. Plates were kept overnight at 37°C. Each condition was performed in duplicate. *Day 2.* For each well, pictures were taken by light and fluorescent microscopy using a 4x objective. To count CFSE-labeled invading tumor cells in the lower chamber, images were taken at the end of co-culture focusing on MSCs at 4x magnification. Images of CFSE-labeled tumor cells invading the matrigel membrane were taken after the co-culture following four washes (in PBS 1x) of the matrigel-coated inserts to allow the removal of non-invading tumor cells from the top of the gel. Inserts were then transferred in new 24 well-plates prefilled with PBS 1x and images taken adjusting the focus on 8µm pores at the bottom of the insert at 4x magnification.

N-MSC overexpression of selected genes

Genes were cloned into a lentiviral plasmid (pLIV) derived from the pLVTH backbone (addgene) to express the puromycin resistance gene (Puro-r). We first cloned the *Emerald* gene (Invitrogen) using a synthetized gBlock DNA fragment (IDT, sequence optimized for synthesis) after the sequence of Puro-r gene and of the self-cleaving P2A peptide (GGAAGCGGAGCTACTAACTTCAGCCTGCTGAAGCAGGCTGGAGACGTGGAGGAGAACCCTGGACCT) (Kim et al., 2011). Briefly, the cloning was done by cutting the pLIV backbone with ClaI and BstEII restriction enzymes (NEB) and using the In-Fusion HD Cloning kit (Clontech, Takara). The resulting pLIV_Puro_2A_Em backbone was used for subsequent cloning of selected genes: *GREM1*, *LOXL2*, *ITGA11* and *ADAMTS12*.

For *GREM1*, we used a single gBlock containing the coding sequence (cds) (NM_013372) and the V5 tag sequence (GGTAAGCCTATCCCTAACCCTCTCCT). For *LOXL2*, two DNA gBlock fragments were synthetized containing the gene cds (NM_002318; optimized for synthesis) and a 3xFlag tag (GGAGATTACAAGGATGACGACGATAAGGGCGACTACAAAGACGATGACGACAAAGGCGATTACAAAGATGACGATGACAAGGGCTAA). V5-tagged *ITGA11* cds (NM_001004439) was synthetized using three gBlock DNA fragments. For *ADAMTS12*, four fragments were synthetized containing the cds (NM_030955) and a 3XFlag tag. All tag sequences were added at the C-terminal of the proteins. After pLIV_Puro_2A_Em digestion with EcoRI restriction enzyme (NEB), the Gibson Assembly Master Mix (ref. E2611, NEB) was used to assemble DNA fragments and backbone.

For *ADAMTS12*, we amplified by PCR the ADAMTS12-3xFlag sequence from the Gibson Assembly reaction using the Phusion High-Fidelity DNA Polymerase (NEB) and the following primers: Forward ACTATAGGGCGGTTTAAACTGTTCGAAACGAATTCGCCACCATGCCATGTGCCCAGAG; Reverse ATTATCATAGGATCCATCATATGAGACGCGTTTAGCCCTTGTCATCGT. The resulting PCR product was then cloned using the TOPO Blunt PCR cloning kit (Invitrogen) in the pLIV_Puro_2A_Em backbone after MluI and BstBI (NEB) digestion and ligation by T4 DNA ligase (NEB).

Infection of primary N- and T-MSCs from patient 26 was performed at passage 5 using lentiviruses at a 0.5 MOI (multiplicity of infection). For viral production HEK 293T packaging cells were transfected with the plasmid of interest, GAG/POL and VSV using FuGene HD (Promega). For *GREM1* and *LOXL2* overexpression, virus containing media were harvested after 72 hours, 0.45µm filtered (Millipore) and ultracentrifuged for 2 hours at 19'500 RPM using a SW28 rotor (Beckman Coulter). Supernatants were decanted and pellets were resuspended by gentle pipetting. For *ITGA11* and *ADAMTS12*, virus containing media were concentrated using the Lenti-X Concentrator (Clontech)

following manufacturer instructions. Concentrated virus were then frozen at -80°C and titrated by FACS (after thawing) looking at the proportion of Emerald positive cells among infected MSCs to assess the MOI.

Gene correlation

Correlation heatmap. Mutual correlation between the four selected genes (*GREM1*, *LOXL2*, *ITGA11* and *ADAMTS12*) was assessed in different microarray datasets from non-small cell lung carcinoma primary tumor samples available on R2 genomics site (https://hgserver1.amc.nl/cgi-bin/r2/main.cgi) using the analysis “found correlating genes with single gene”. Here we showed genes correlated with *GREM1* (218469_at probe) from the dataset “Tumor Non-small cell lung carcinoma - Plamadeala - 410 - MAS5.0 - u133p2” (GEO ID: GSE63074) with a R p-value cutoff of 1E-53.

Correlation matrix. To create the correlation matrix in Figure S3, expression profiling by array data of 16 carcinoma-associated fibroblasts (CAFs) were downloaded from GSE22874. Expression profiling by array data of 5 mesenchymal stem cells derived from non-small cell lung cancer (NSCLC-MSCs) were downloaded from GSE23066. Correlation and p-values within with two datasets and our data (T-MSCs in lung carcinoma) were computed with R function cor() with default parameters.

Survival curves

Kaplan-Meier survival plots (overall survival) for patients with non-small cell lung cancer were obtained from transcriptome data (mRNA) from www.kmplot.com/lung (Győrffy et al., 2013). For the selected genes (selected probe is reported), patients were split by medians auto-selecting the best cutoff. Data from the 2015 version database were analyzed using a univariate Cox regression and excluding biased arrays. For each graph, hazard ratio (HR) and log-rank p-value are reported. A graph with the mean expression of the 4 selected probes was also created using the multigene classifier function and giving the same weight to each probe.

Supplemental References

Benjamini, Y., Hochberg, Y. (1995). Controlling the False Discovery Rate: A Practical and Powerful Approach to Multiple Testing. J. R. Stat. Soc. Ser. B *57*, 289–300.

Cox, J., Mann, M. (2008). MaxQuant enables high peptide identification rates, individualized p.p.b.-range mass accuracies and proteome-wide protein quantification. Nat. Biotechnol. *26*, 1367–1372.

Cox, J., Neuhauser, N., Michalski, A., Scheltema, R.A., Olsen, J. V., Mann, M. (2011). Andromeda: A Peptide Search Engine Integrated into the MaxQuant Environment. J. Proteome Res. *10*, 1794–1805.

Győrffy, B., Surowiak, P., Budczies, J., Lánczky, A., Higashiyama, M. (2013). Online Survival Analysis Software to Assess the Prognostic Value of Biomarkers Using Transcriptomic Data in Non-Small-Cell Lung Cancer. PLoS One *8*, e82241.

Jain, N., Thatte, J., Braciale, T., Ley, K., O'Connell, M., Lee, J.K. (2003). Local-pooled-error test for identifying differentially expressed genes with a small number of replicated microarrays. Bioinformatics *19*, 1945–51.

Kim, J.H., Lee, S.-R., Li, L.-H., Park, H.-J., Park, J.-H., Lee, K.Y., Kim, M.-K., Shin, B.A., Choi, S.-Y. (2011). High Cleavage Efficiency of a 2A Peptide Derived from Porcine Teschovirus-1 in Human Cell Lines, Zebrafish and Mice. PLoS One *6*, e18556.

Mauvoisin, D., Wang, J., Jouffe, C., Martin, E., Atger, F., Waridel, P., Quadroni, M., Gachon, F., Naef, F. (2014). Circadian clock-dependent and -independent rhythmic proteomes implement distinct diurnal functions in mouse liver. Proc. Natl. Acad. Sci. U. S. A. *111*, 167–72.

Müller, A.M., Nesslinger, M., Skipka, G., Müller, K.-M. (2002). Expression of CD34 in Pulmonary Endothelial Cells in vivo. Pathobiology *70*, 11–7.

Shannon, P., Markiel, A., Ozier, O., Baliga, N.S., Wang, J.T., Ramage, D., Amin, N., Schwikowski, B., Ideker, T. (2003). Cytoscape: a software environment for integrated models of biomolecular interaction networks. Genome Res. *13*, 2498–504.

Szklarczyk, D., Franceschini, A., Wyder, S., Forslund, K., Heller, D., Huerta-Cepas, J., Simonovic, M., Roth, A., Santos, A., Tsafou, K.P., et al. (2015). STRING v10: protein-protein interaction networks, integrated over the tree of life. Nucleic Acids Res. *43*, D447-52.
